# CMV serostatus is associated with improved survival and delayed toxicity onset following anti-PD-1 checkpoint blockade

**DOI:** 10.1038/s41591-025-03647-1

**Published:** 2025-04-23

**Authors:** Gusztav Milotay, Martin Little, Robert A. Watson, Dylan Muldoon, Sophie MacKay, Ayako Kurioka, Orion Tong, Chelsea A. Taylor, Isar Nassiri, Louisa M. Webb, Oluwafemi Akin-Adigun, Julia Bremke, Weiyu Ye, Bo Sun, Piyush Kumar Sharma, Ros Cooper, Sara Danielli, Flavia Matos Santo, Alba Verge de Los Aires, Guangyi Niu, Lea Cohen, Esther Ng, James J. Gilchrist, Amanda Y. Chong, Alex Mentzer, Victoria Woodcock, Nicholas Coupe, Miranda J. Payne, Michael Youdell, Mark R. Middleton, Paul Klenerman, Benjamin P. Fairfax

**Affiliations:** 1 MRC Weatherall Institute of Molecular Medicine, University of Oxford, Oxford, UK; 2 Department of Oncology, University of Oxford, Oxford, UK; 3 Oxford Cancer—CRUK Oxford Centre, University of Oxford, Churchill Hospital, Oxford, UK; 4 Kennedy Institute for Rheumatology, University of Oxford, Oxford, UK; 5 The Centre for Human Genetics, University of Oxford, Oxford, UK; 6 Translational Gastroenterology Unit, John Radcliffe Hospital, Oxford, UK; 7 Nuffield Department of Clinical Neuroscience, University of Oxford, Oxford, UK; 8 Department of Paediatrics, University of Oxford, Oxford, UK; 9 Big Data Institute, Li Ka Shing Centre for Health Information and Discovery, University of Oxford, Oxford, UK; 10 Cancer and Haematology Centre, Oxford University Hospitals NHS Foundation Trust, Oxford, UK; 11 NIHR Oxford Biomedical Research Centre, Oxford University Hospitals NHS Foundation Trust, Oxford, UK

**Keywords:** Melanoma, Cancer immunotherapy, Tumour immunology, Infection

## Abstract

Cytomegalovirus (CMV) is a globally endemic latent herpes virus that profoundly impacts T cell immunity. We investigated the oncological consequences of CMV infection across 341 prospectively recruited patients receiving immune checkpoint blockade (ICB) for melanoma. CMV^+^ patients with metastatic melanoma (MM) have higher lymphocyte counts, reduced neutrophil to lymphocyte ratio and divergent CD8^+^ T cell gene expression. Combination anti-CTLA-4/anti-PD-1 ICB, but not single-agent anti-PD-1 ICB, induces cytotoxicity and CMV-associated gene expression in CD8^+^ T cells from CMV^−^ patients. Correspondingly, overall survival was independent of CMV serostatus in combination anti-CTLA-4/anti-PD-1 ICB recipients (CMV^+^ hazard ratio for death: 1.02, *P* = 0.92), whereas CMV^+^ single-agent anti-PD-1 ICB recipients had improved overall survival (CMV^+^ hazard ratio for death: 0.37, *P* < 0.01), a finding also seen in CMV^+^ adjuvant single-agent anti-PD-1 ICB recipients (CMV^+^ hazard ratio for recurrence: 0.19, *P* = 0.03). We identify *TBX21*, encoding T-bet, as a transcriptional driver of CMV-associated CD8^+^ T cell gene expression, finding that *TBX21* expression is predictive of overall survival (hazard ratio: 0.62, *P* = 0.026). CMV^+^ patients unexpectedly show reduced cumulative incidence of grade 3+ immune-related adverse events at 6 months (0.30 versus 0.52, *P* = 2.2 × 10^−5^), with lower incidence of colitis (*P* = 7.8 × 10^−4^) and pneumonitis (*P* = 0.028), an effect replicated in non-melanoma ICB recipients (*n* = 58, *P* = 0.044). Finally, we find reduced CMV seropositivity rates in patients with MM compared with UK Biobank controls (odds ratio: 0.52, *P* = 1.8 × 10^−4^), indicating CMV seropositivity may protect against MM. Specifically, patients with *BRAF*-mutated MM are less likely to be CMV^+^ (odds ratio = 2.2, *P* = 0.0054), while CMV^−^ patients present 9 yr earlier with *BRAF* wild-type MM (*P* = 1.3 × 10^−4^). This work reveals an interaction between CMV infection, MM development according to *BRAF* status and response to ICB, while demonstrating CMV infection is protective against severe ICB immune-related adverse events, highlighting the potential importance of previous infection history and chronic immune activation in MM development and immunotherapy outcomes.

## Abstract (web summary)

Analysis of multiple cohorts of patients with melanoma demonstrates a positive association between cytomegalovirus serostatus and overall survival in patients treated with monotherapy but not combination immune checkpoint blockade, as well as delayed onset of immune-related adverse events across both treatment types, as well as delayed development of metastatic disease in seropositive patients.

## Main

Tumor infiltrating T cells commonly do not cross-react with cancer-related antigens. Instead, viruses form a major source of putative antigens for these ‘bystander’ T cells^1–3^. The anti-tumorigenic properties of bystander T cells and their role in tumor development remains unresolved. CMV^4,5^ (Human Herpes Virus 5, HHV5), a betaherpes virus that causes latent, chronic viral infection (CVI) with spontaneous reactivation^6^, forms a key source of bystander T cell-reactive viral antigens. Global seroprevalence of CMV infection is high, although seropositivity varies between countries and is associated with sociodemographic factors including population density^7,8^. Analysis of UK Biobank (UKB) participants found 56.1% of white British aged 40–70 are CMV seropositive (CMV^+^), with seropositivity rising by approximately 0.7% per year from just under 50% aged 40 (ref. ^9^). CMV infection is a cause of serious morbidity in neonates and immunocompromised individuals, while in otherwise healthy people, health associations include increased cardiovascular disease rates and incident mortality^10^. These observations are controversial however, and a recent study in the UKB did not demonstrate causality with cardiovascular disease^11^. Most notably, CMV elicits the induction of memory T cell inflation. This consists of durable hyper-expansion of effector memory T cells re-expressing CD45RA (TEMRA) that display preserved effector function^12–14^, with consequent skewing of the clonal repertoire^15^. In the context of MM we have shown that increased numbers of large CD8^+^ T cell clones both before and after ICB are associated with improved overall survival (OS)^16,17^. The relationship between CMV infection (defined as IgG-reactive seropositivity) and ICB response in melanoma has not been methodically examined, however. Therefore, given the prevalence of CMV-reactive bystander clones and the correspondence between increased T cell clonality and CMV infection, we have systematically assessed links between CMV seropositivity and ICB outcomes for melanoma, analyzing data from prospectively recruited patients for whom CMV serology could be determined in the Oxford Cancer Immunotherapy Toxicity and Efficacy (OxCITE) cohort study.

## CMV and pretreatment hematological and immune profiles

**Fig. 1:**
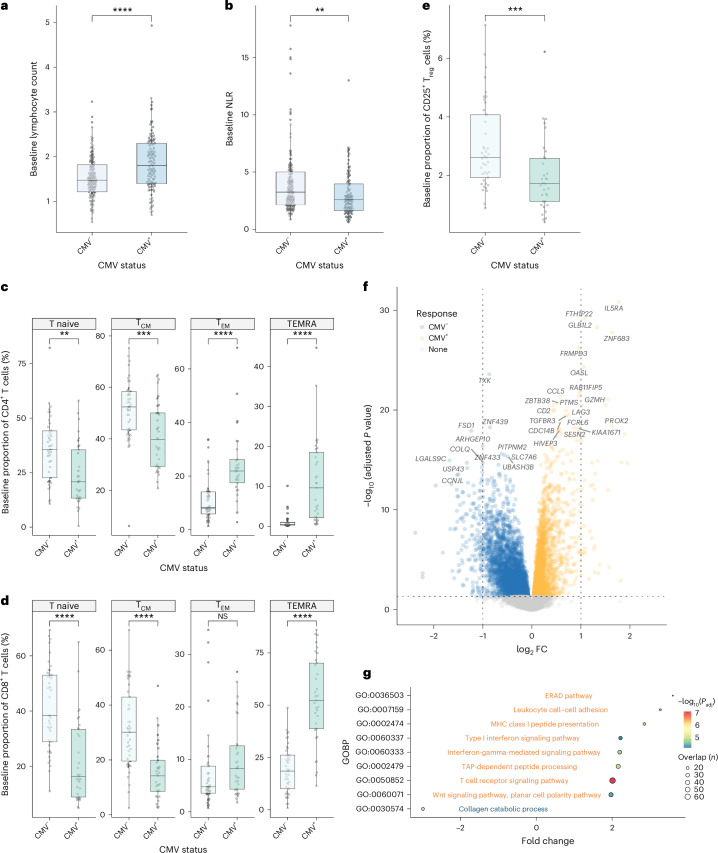
Baseline hematological and immunological associations of CMV infection in patients with melanoma. **a**, Pretreatment lymphocyte count is elevated in CMV^+^ patients with MM (*n* = 124 CMV^−^, *n* = 105 CMV^+^; *P* = 9.1 × 10^−5^; lower and upper box hinges represent the 25th to 75th percentiles, the central line represents the median and the whiskers extend to the highest and lowest values no greater than 1.5 × IQR). **b**, CMV pretreatment NLR is reduced in CMV^+^ patients (*P* = 0.0015; samples and boxplot as in **a**). **c**, Flow cytometry-derived pretreatment T cell subset proportions according to CMV serostatus for CD4^+^ T cells (*n* = 41 CMV^−^, *n* = 33 CMV^+^; *P* = 0.0010 T naive, *P* = 7.3 × 10^−4^ T_CM_, *P* = 5.2 × 10^−6^ T_EM_, *P* = 6.6 × 10^−9^ TEMRA; boxplot as in **a**). **d**, CD8^+^ T cells (*P* = 1.8 × 10^−6^ T naive, *P* = 3.2 × 10^−6^ T_CM_, *P* = 0.068 T_EM_, *P* = 1.6 × 10^−9^ TEMRA; samples and boxplots as in **c**). **e**, Baseline pretreatment CD25^+^FOXP3^+^CD4^+^ T cells according to CMV serostatus (*P* = 7.6 × 10^−4^; samples and boxplots as in **c**). **f**, Differentially expressed genes (DEGs) according to CMV serostatus from pretreatment CD8^+^ T cells; *y* axis shows Benjamini–Hochberg-corrected −log_10_(*P*_adj_) derived from negative binomial Wald test using CMV^−^ samples as a reference (*n* = 111 CMV^−^, *n* = 95 CMV^+^). **g**, Gene Ontology Biological Process (GOBP) analysis of DEGs from **f**, highlighting induction/suppression (orange and blue, respectively) of pathways involved in T cell activation in CMV seropositivity. All statistical tests were two-sided Wilcoxon rank sum unless otherwise stated. FC, fold-change; NS, not significant; T_CM_, central memory T cells. Source data

**Extended Data Fig. 1:**
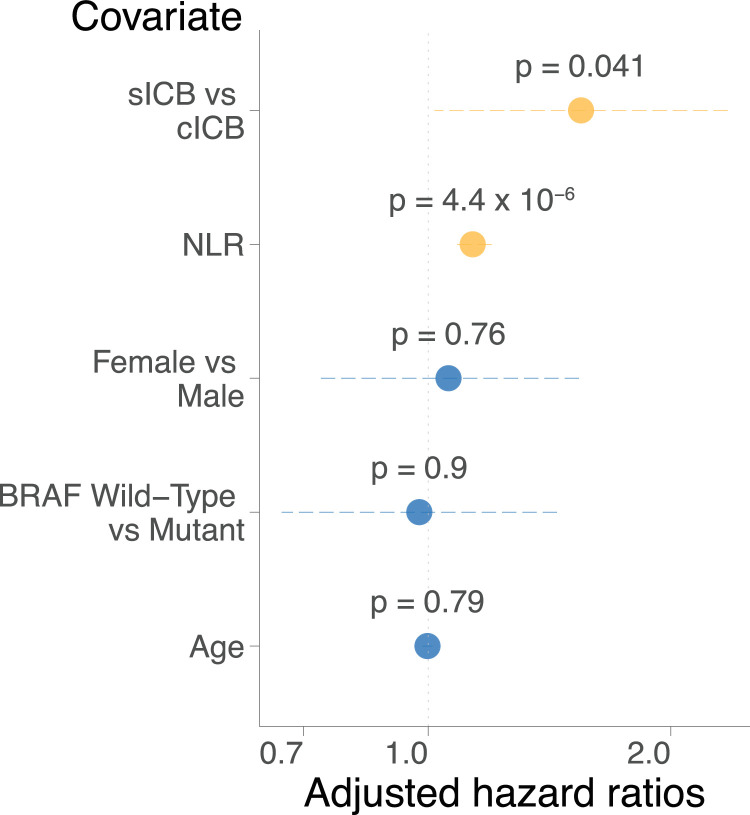
Increased neutrophil:lymphocyte ratio is negatively prognostic in metastatic melanoma. Cox proportional hazards model of covariates including neutrophil:lymphocyte ratio and occurrence of death in patients with MM (n = 258, HR_adj_ = 1.1, CI 1.08:1.2, P = 4.4 × 10^−6^, Wald test). Source data

We assessed 341 patients within the OxCITE study who had received ICB as standard-of-care treatment for unresectable/metastatic melanoma (MM, 302 of 341) or in the adjuvant setting for resected stage II/III/IV melanoma (Adjuvant, 39 of 341), for whom 308 had pretreatment samples for CMV serotyping (Extended Data Table 1). Full blood counts assessed before treatment with single-agent anti-PD-1 ICB (sICB) or combination anti-CTLA-4/anti-PD-1 ICB (cICB) for unresectable/metastatic melanoma demonstrated CMV seropositivity was associated with increased baseline lymphocyte count (*n* = 229; 124 CMV^−^, median = 1.5 × 10^9^ l^−1^; 105 CMV^+^, median = 1.8 × 10^9^ l^−1^; difference in interval −0.30 × 10^9^ l^−1^; 95% confidence interval (95% CI), −0.46 to −0.15; *P* = 9.1 × 10^−5^ Wilcoxon rank sum test; Fig. 1a). We explored the relationship between CMV serostatus and the neutrophil to lymphocyte ratio (NLR), a melanoma prognostic marker^18^. Consistent with the negative prognostic association of a raised NLR, increasing NLR in OxCITE patients with MM was a hazard for death, the other key significant clinical factor identified being sICB treatment (Extended Data Fig. 1). Interestingly, CMV seropositivity was associated with a significantly reduced NLR (124 CMV^−^, median = 3.3; 105 CMV^+^, median = 2.6; interval = 0.68; 95% CI, 0.26 to 1.1; *P* = 0.0015 Wilcoxon rank sum test; Fig. 1b), indicative of a favorable pretreatment immune profile. To develop a more granular picture of the immuno-modulatory consequences of CMV in patients, flow cytometry was performed on pretreatment peripheral blood mononuclear cells (PBMCs) (*n* = 74), assessing CD4^+^ and CD8^+^ T cell subsets. Consistent with observations in healthy individuals^14^, CMV seropositivity is associated with skewing of patient pretreatment T cell subsets, with naive and central memory subset depletion and expansion of effector memory (T_EM_) and TEMRA subsets (Fig. 1c,d). Patients with CMV also had significantly fewer circulating FOXP3^+^CD25^+^ regulatory T cells (T_reg_ cells) (*P* = 7.6 × 10^−4^; Fig. 1e), which are negatively prognostic in melanoma^19^ and form a target for anti-CTLA-4-mediated antibody-dependent cellular cytotoxicity^20^. Given their key role in mediating ICB response, we focused on CD8^+^ T cells, exploring the pretreatment transcriptomic effects of CMV serostatus across patients with MM with available serology data. Samples taken before first ICB treatment from 206 patients with MM (95 CMV^+^, 111 CMV^−^) demonstrated profound divergence in CD8^+^ gene expression according to CMV serostatus, with differential expression of 7,576 of 17,308 transcripts (*P*_adj_ < 0.05, 19% of all transcripts upregulated, 25% downregulated; Fig. 1f and Supplementary Table 1). CMV-induced transcripts consisted of many encoding cell-surface proteins implicated in T cell signaling, including *CD2*, associated with enhanced chimeric antigen receptor T-cell (CAR-T) activity^21^, *LAG3*, the target of relatlimab^22^, and *CD8A*, a T cell co-receptor. We additionally observed upregulation of *ZNF683*, encoding a transcription factor (TF) found to be induced in HCMV-reactive T cells^23,24^, and latterly implicated in effector/resident memory T cells involved in CD8^+^ T cell anti-PD1 response^25,26^. Gene ontology analysis highlighted differential modulation of 181 pathways (141 induced, 40 downregulated; Fig. 1g and Supplementary Table 2) involving key CD8^+^ T cell functions. Notably, CMV seropositivity was associated with upregulation of T cell receptor (TCR) signaling (*P*_adj_ = 8.2 × 10^−8^), interferon γ (IFNγ)-mediated signaling (*P*_adj_ = 1.8 × 10^−5^) and antigen processing and presentation via class I Major Histocompatibility Complex (*P*_adj_ = 1.1 × 10^−5^), all crucial to anti-cancer immunity.

## CMV serostatus alters transcriptomic and clinical response to ICB

**Fig. 2:**
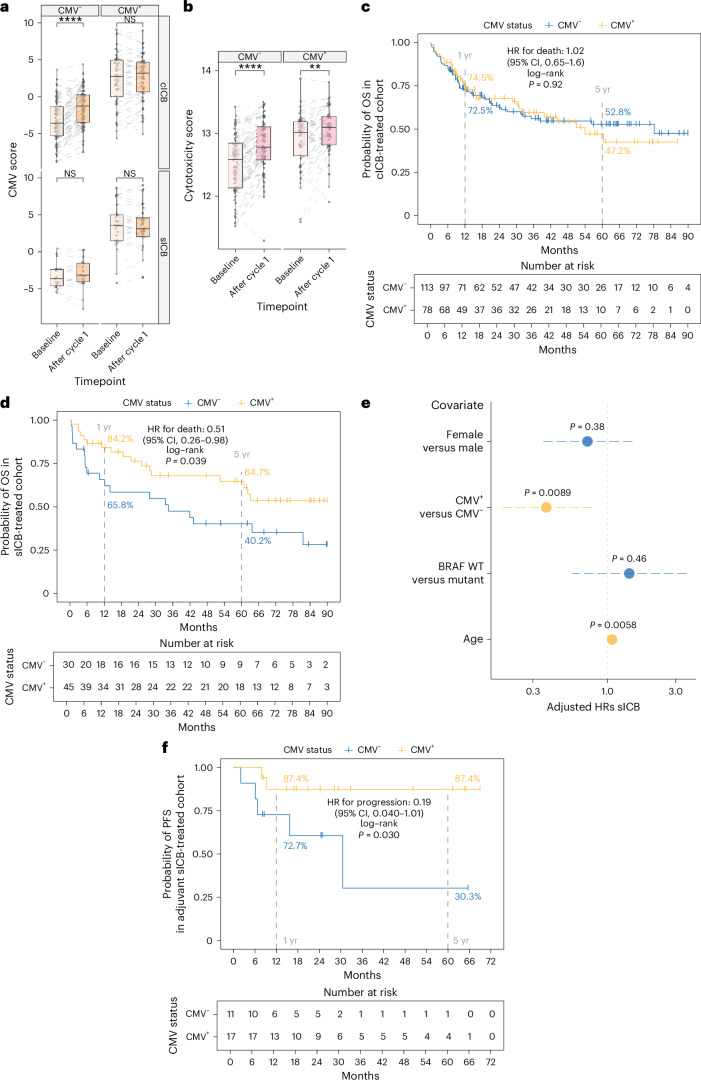
CMV is associated with divergent CD8^+^ T cell transcriptomic and clinical response to ICB. **a**, cICB treatment of CMV^−^ patients leads to a highly significant induction of CMV score (*y* axis) (top-left panel), not observed in CMV^−^ patients receiving sICB (bottom-left panel) (*n* = 79 CMV^−^, *n* = 72 CMV^+^; *P* = 2.8 × 10^−9^ cICB CMV^−^, *P* = 0.28 sICB CMV^−^, *P* = 0.41 cICB CMV^+^, *P* = 0.54 sICB CMV^+^). *P* values derived from two-sided Wilcoxon signed-rank test; lower and upper box hinges represent the 25th to 75th percentiles, the central line represents the median and the whiskers extend to the highest and lowest values no greater than 1.5 × IQR. **b**, cICB treatment leads to a significant induction of cytotoxicity score irrespective of CMV status (*n* = 64 CMV^−^ *P* = 2.1 × 10^−7^, *n* = 44 CMV^−^ *P* = 0.0013). *P* values derived from two-sided Wilcoxon signed-rank test; boxplot as in **a**. **c**, Kaplan–Meier analysis shows no significant difference in OS of cICB-treated CMV^+^ and CMV^−^ patients (*n* = 113 CMV^−^, *n* = 78 CMV^+^; *P* = 0.92, two-sided log-rank test). **d**, Kaplan–Meier analysis demonstrates increased OS of CMV^+^ versus seronegative sICB recipients (*n* = 30 CMV^−^, *n* = 45 CMV^+^; *P* = 0.039, two-sided log-rank test). **e**, Cox proportional hazards model as per **d** shows significantly reduced adjusted HR for death in CMV^+^ recipients of sICB (HR_adj_ = 0.37; 95% CI, 0.18 to 0.78; *P* = 0.0089, Wald test). Increased age is significantly associated with increased risk of death when controlling for CMV (HR_adj_ = 1.07; 95% CI, 1.02 to 1.13; *P* = 0.0058, Wald test). **f**, Improved PFS in stage II/III resectable patients following sICB in CMV^+^ patients relative to CMV^−^ patients (*n* = 11 CMV^−^, *n* = 17 CMV^+^; *P* = 0.030, two-sided log-rank test). Source data

**Extended Data Fig. 2:**
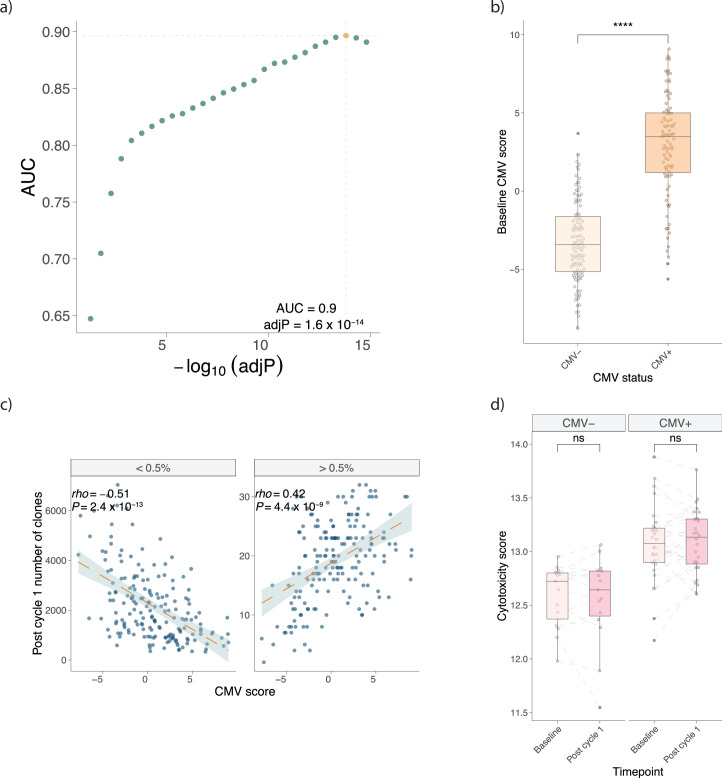
Derivation of CMV score. (**a**) ROC analysis of CMV seropositivity prediction using the first principal component of DEGs between baseline CD8^+^ samples based on CMV status. Area under the curve (y axis) is plotted against the Benjamini-Hochberg-corrected -log_10_(adjusted P value) threshold (x axis) used to determine genes to be incorporated in the score with the optimal threshold labelled. (**b**) CMV score of pre-treatment patient CD8^+^ T-cell samples according to CMV seropositivity (n = 111 CMV-, n = 95 CMV+, P < 2.2 × 10^−16^, two-sided Wilcoxon rank sum test); lower and upper box hinges represent the 25th to 75th percentiles, the central line represents the median and the whiskers extend to the highest and lowest values no greater than 1.5 × interquartile range. (**c**) Correlation of CMV score with number of CD8^+^ clones stratified by repertoire occupancy post one cycle of immunotherapy (n = 180, Spearman’s Rank Correlation test, rho denotes the Spearman rho, and P value is from two-sided t-test). (**d**) Cytotoxicity score is not induced following sICB, irrespective of CMV status (n = 43, CMV- P = 0.93, CMV + P = 0.58, two-sided Wilcoxon signed-rank test; boxplot as in (**b**)). Source data

Given the association of CMV seropositivity with favorable pretreatment hematological indices and the divergence in baseline CD8^+^ T cell gene expression according to CMV status, we explored the effect of ICB treatment on CMV-associated gene expression. To capture the CMV phenotype in a single value, we developed a CMV transcriptomic score based on the first principal component of differentially expressed genes that yielded a maximal area under the curve (AUC) for CMV serostatus prediction from CD8^+^ T cell bulk RNA sequencing (RNA-seq) (75 genes providing an AUC of 0.90; Extended Data Fig. 2a). Correspondingly, pretreatment samples showed a significant difference in CMV score according to serostatus (Extended Data Fig. 2b). Furthermore, the CMV score was associated with the proportional composition of TCR within the transcriptome—a surrogate for clonal size—with a significant positive correlation between CMV score and number of clones occupying >0.5% of the clonal repertoire (Extended Data Fig. 2c). The CD8^+^ transcriptional response differs substantially between sICB and cICB treatments^16^, and hence the interaction between CMV status and transcriptomic response was independently assessed for each treatment. While in CMV^+^ patients no effect of either sICB or cICB on the CMV score was observed, in CMV^−^ patients the response varied according to ICB type. Patients receiving cICB demonstrated a significant increase in CMV score (*P* = 2.8 × 10^−9^, Wilcoxon signed-rank test), whereas in sICB recipients the CMV score did not significantly change (*P* = 0.28; Fig. 2a). Similarly, expression of cytotoxic genes correlated with IFNγ—corresponding to a previously described predictive cytotoxicity score^17^ (Fig. 2b and Supplementary Table 3)—was induced in CMV^−^ cICB recipients, but not in sICB recipients (Extended Data Fig. 2d). This analysis demonstrates cICB treatment leads to partial transcriptomic convergence of CD8^+^ T cell gene expression between CMV^+^ and CMV^−^ patients which is not seen in sICB recipients, indicating the immunological response to ICB in MM depends upon both the patient CMV status and the addition of anti-CTLA-4 treatment. To explore clinical implications of these observations, Kaplan–Meier analyses of OS in patients receiving ICB for metastatic/unresectable melanoma with a minimum of 6 months of follow-up after treatment initiation were performed. Within the patients receiving cICB, there was no difference in median OS according to CMV serostatus (*n* = 191; median survival 113 CMV^−^: 78 months, 78 CMV^+^: 54 months; hazard ratio (HR) = 1.02; *P* = 0.92, log-rank test; Fig. 2c). Analysis of sICB-treated patients, however, demonstrated that CMV^+^ patients had significantly prolonged OS compared with CMV^−^ patients (*n* = 75; 30 CMV^−^, median OS 34.4 months; 45 CMV^+^, median OS not reached; HR = 0.51; *P* = 0.039, log-rank test; Fig. 2d), which was robust in multivariable analysis (HR = 0.37, *P* = 0.0089; Fig. 2e). Notably, multivariable analysis highlighted that when controlling for CMV, age at systemic treatment initiation becomes a significant risk factor for death (*P* = 0.0058), consistent with evidence that the effects of CMV are distinct to those of age-associated immunosenescence^27^. To further explore the relationship between CMV infection and outcomes following sICB, we analyzed a separate group of prospectively recruited stage II/III patients receiving adjuvant sICB after complete-resection. Again, we found CMV^+^ recipients of sICB had improved prognosis, demonstrating a significantly improved recurrence-free survival (HR for recurrence = 0.19, *P* = 0.030; Fig. 2f). In sum, these results support CMV seropositivity conferring induction of a prognostically favorable CD8^+^ T cell gene set that is also invoked by cICB but not sICB, resulting in divergent outcomes in sICB recipients according to CMV serostatus.

## CMV^+^ patients have fewer severe immune-related adverse events

**Fig. 3:**
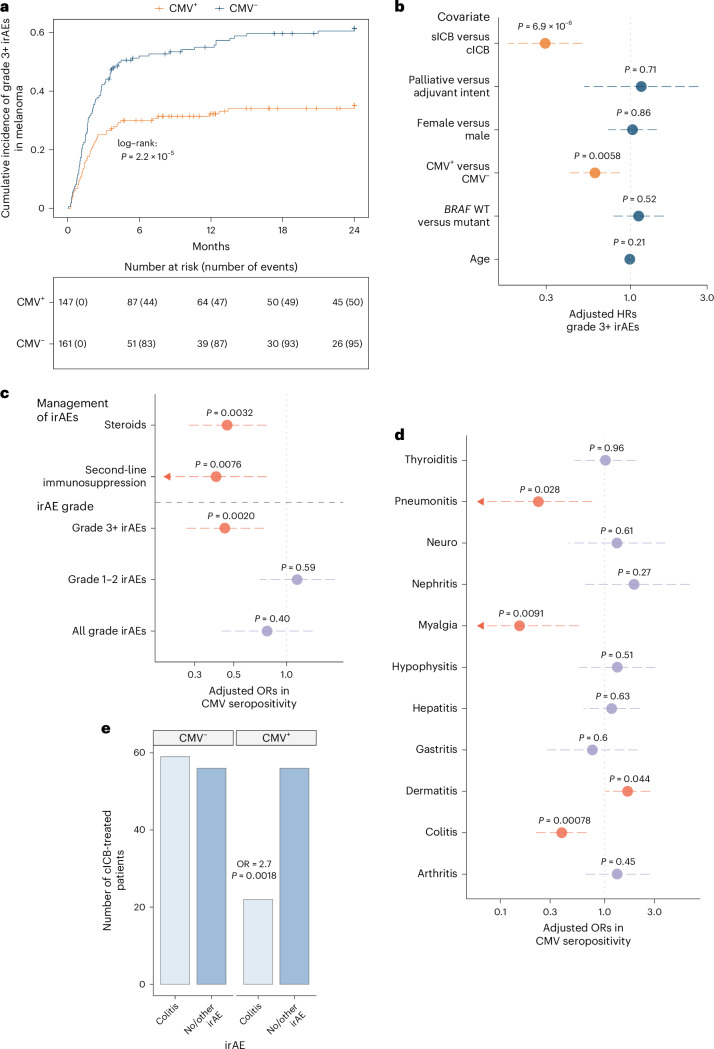
CMV is associated with organ-specific irAE risk. **a**, Cumulative incidence of developing grade 3+ irAEs in all ICB-treated patients with melanoma is significantly reduced in CMV seropositivity (*n* = 161 CMV^−^, *n* = 147 CMV^+^; *P* = 2.2 × 10^−5^, two-sided log-rank test). **b**, Multivariable semi-competing risks analysis, where death is a semi-competing risk, indicates that CMV is protective against grade 3+ irAE development when adjusting for age, sex, treatment type, *BRAF* mutation status and treatment intent as covariates (HR_adj_ = 0.60; 95% CI, 0.42 to 0.86; *P* = 0.0058, Wald test; patients as in **a**). **c**, Time-independent adjusted ORs for development of all grade, grade 3+ and grade 1–2 irAEs and requirement of steroid or second-line immunosuppression in CMV^+^ patients, adjusting for covariates as per **b** (binary logistic regression) (steroids: OR_adj_ = 0.46; 95% CI, 0.27 to 0.77; *P* = 0.0032; second-line immunosuppressants: OR_adj_ = 0.40; 95% CI, 0.20 to 0.77; *P* = 0.0076; grade 3+ irAEs: OR_adj_ = 0.45; 95% CI, 0.27 to 0.74; *P* = 0.0020; patients as in **a**). **d**, Adjusted ORs for development of all grade organ-specific irAEs in CMV^+^ patients (binary logistic regression) adjusting for covariates as per **b** (colitis: OR_adj_ = 0.39; 95% CI, 0.22 to 0.67; *P* = 7.8 × 10^−4^; pneumonitis: OR_adj_ = 0.23; 95% CI, 0.060 to 0.76; *P* = 0.028; myalgia: OR_adj_ = 0.15; 95% CI, 0.060 to 0.56; *P* = 0.0091; dermatitis: OR_adj_ = 1.66; 95% CI, 1.02 to 2.7; *P* = 0.044; patients as in **a**). **e**, CMV^+^ cICB-treated patients are at significantly reduced odds of developing colitis (*n* = 115 CMV^−^, *n* = 78 CMV^+^; two-sided Fisher’s exact test, OR = 2.7; 95% CI, 1.4 to 5.2; *P* = 0.0018). Source data

**Extended Data Fig. 3:**
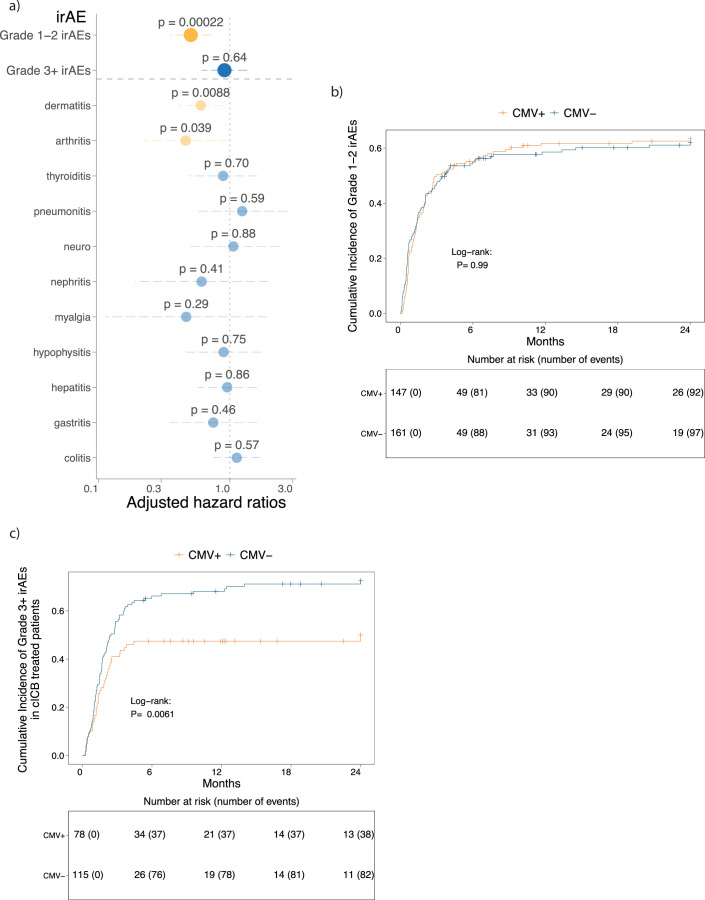
Relationship between organ-specific toxicity development and HR for death. (**a**) Cox proportional hazards analysis of irAEs by grade and by organ specificity demonstrates grade 1-2 irAEs and specifically dermatitis and arthritis bestow reduced risk of death in MM (n = 299, Dermatitis: HR_adj_ = 0.60, CI 0.41:0.88, P = 0.0088; Arthritis: HR_adj_ = 0.46, CI 0.22:0.96, P = 0.039; Wald test). (**b**) Cumulative incidence of grade 1-2 irAEs according to CMV status in melanoma (n = 161 CMV-, n = 147 CMV+; P = 0.99, two-sided log-rank test). (**c**) Cumulative incidence of grade 3+ irAEs is significantly lower in CMV+ patients once restricting to cICB treatment (n = 115 CMV-, n = 78 CMV+; P = 0.0061, two-sided log-rank test). Source data

Immune-related adverse events (irAEs) are a frequent complication of ICB treatment, with grade 3+ irAEs, which represent the most clinically consequential toxicities, observed in real-world clinical data in 10–15% of sICB recipients and ~60% of cICB recipients^28–30^. While irAEs are a cause of morbidity and increased demand on healthcare resources, multiple studies have reported associations between irAE development and improved survival in patients receiving ICB for cancer^28,31,32^. Using a time-dependent Cox-regression model to account for guarantee–time bias susceptibility, we assessed the relationship between the irAE development and clinical outcome in ICB-treated MM^33^. Analysis at the level of organ-specific irAEs demonstrated that, as per other studies, both arthritis and dermatitis were associated with improved OS^34^. Summarizing across irAEs showed that mild grade 1–2 irAEs were significantly associated with improved survival, whereas grade 3+ irAEs were not (Extended Data Fig. 3a). This is in keeping with meta-analyses and may reflect the use of steroids and other immunosuppressants in high-grade irAE management potentially antagonizing the anti-cancer effect of ICB^31,35,36^. Given our findings, we assessed the influence of CMV on irAE development, estimating cumulative incidence and time to irAEs using a univariate semi-competing risks model (death being the competing risk) across all patients with melanoma treated with anti-PD-1 or anti-PD-1/anti-CTLA-4 (*n* = 308). While no effect of CMV seropositivity was seen with grade 1–2 irAEs (Extended Data Fig. 3b), CMV was associated with delayed time to onset and reduced cumulative incidence of severe grade 3+ irAEs (0.52 versus 0.30 at 6 months, *P* = 2.2 × 10^−5^; Fig. 3a) which was also significant when analysis was confined to cICB-treated patients with MM (Extended Data Fig. 3c). When using multivariable analysis to control for age, sex, ICB type, *BRAF* status and treatment intent, both the known comparative reduced toxicity of sICB and CMV remained independently associated with significantly reduced incidence of grade 3+ irAEs (*P* = 0.0058; Fig. 3b). We then analyzed this CMV association across the entire time-course of patient ICB treatment using binomial logistic regression, again noting no association with grade 1–2 irAEs, but finding a significantly reduced incidence of grade 3+ irAEs (odds ratio (OR) = 0.45, *P* = 0.0020; Fig. 3c). Accordingly, CMV^+^ patients had reduced requirements for steroids (OR = 0.46, *P* = 0.0032) and second-line immunosuppression given upon steroid failure to control toxicity (OR = 0.40, *P* = 0.0076). We subsequently explored whether CMV is associated with organ-specific irAEs across all patients. This showed CMV seropositivity is associated with reduced incidence of distinct irAEs, namely colitis (OR = 0.39, *P* = 7.8 × 10^−4^), pneumonitis (OR = 0.23, *P* = 0.028) and myalgia (OR = 0.15, *P* = 0.0091). We also noted CMV conversely leads to an increased risk of skin irAEs (OR = 1.66, *P* = 0.044), but could not discern other organ-specific effects (Fig. 3d,e). Finally, we explored evidence for CMV protection against colitis, myalgia and pneumonitis in patients receiving ICB for non-melanoma cancer with greater than 3 months of follow-up within OxCITE (total *n* = 58, 26 of 58 CMV^+^; Extended Data Fig. 1b). In total, 0 of 26 CMV^+^ versus 6 of 32 CMV^−^ patients developed one these toxicities within the follow-up window (*P* = 0.044, one-sided Fisher’s exact), suggesting the observed association between CMV and organ-specific irAEs is not melanoma-specific.

## *TBX21* drives CMV-associated CD8^+^ T cell gene expression

**Fig. 4:**
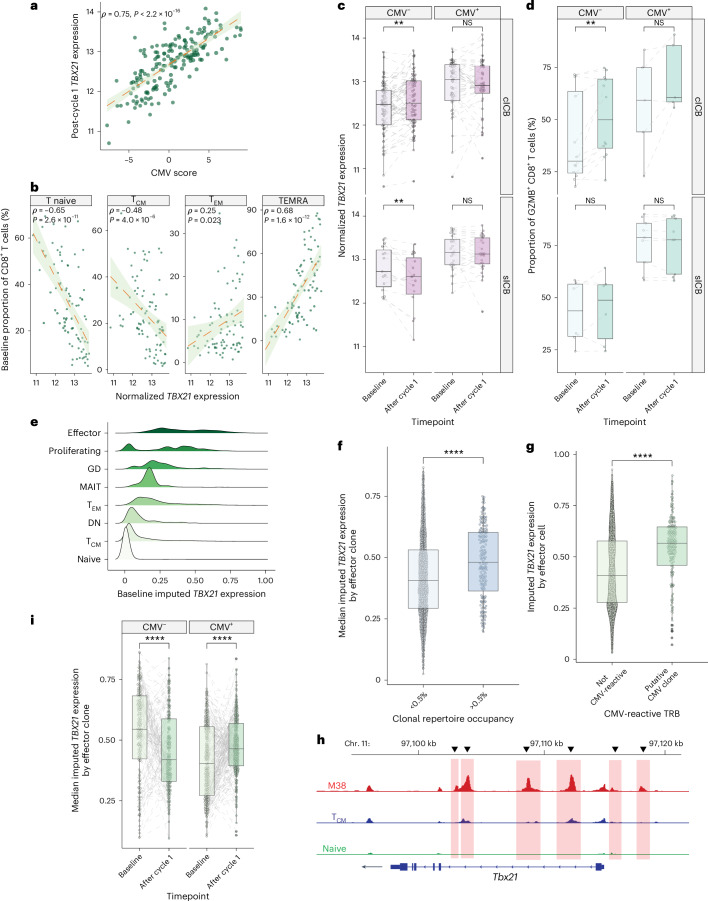
CD8^+^ T cell signature of CMV infection is associated with *TBX21* expression. **a**, Normalized CD8^+^ *TBX21* expression correlated with CMV score after one cycle of immunotherapy (*n* = 181, Spearman’s rank correlation test, *ρ* denotes the Spearman *ρ* and *P* value is from two-sided *t*-test). **b**, Baseline CD8^+^ subset correlations with *TBX21* highlight that increased *TBX21* expression is correlated with increased circulating TEMRA and decreased naive T cell proportions (*n* = 89, Spearman’s rank correlation test, *ρ* and *P* values defined as in **a**). **c**, In CMV^−^ patients, treatment with cICB (top-left panel) is associated with a significant induction of *TBX21* (*n* = 64, *P* = 0.0033). This is not observed in CMV^−^ patients receiving sICB (bottom-left panel) where *TBX21* is significantly downregulated (*n* = 15, *P* = 0.0026). In CMV^+^ patients no significant change in *TBX21* expression is noted in either cICB recipients (top-right panel) (*n* = 44, *P* = 1) or sICB recipients (bottom-right panel) (*n* = 28, *P* = 0.94). All *P* values result from application of a two-sided Wilcoxon signed-rank test; lower and upper box hinges represent the 25th to 75th percentiles, the central line represents the median and the whiskers extend to the highest and lowest values no greater than 1.5 × IQR. **d**, Flow cytometry blinded to CMV and ICB type measuring the proportion of GZMB^+^ CD8^+^ T cells before and after one cycle of ICB, dichotomized by CMV status and ICB type (*n* = 18 CMV^−^, *n* = 14 CMV^+^; cICB CMV^−^: *P* = 0.0024, cICB CMV^+^: *P* = 0.12, sICB CMV^−^: *P* = 0.84, sICB CMV^+^: *P* = 0.44). *P* values relate to a two-sided Wilcoxon signed-rank test; boxplots as in **c**. **e**, Imputed *TBX21* expression by CD8^+^ subsets in sICB-treated patients as assessed using scRNA-seq (*n* = 16). **f**, Median imputed *TBX21* expression by effector clone stratified by peripheral repertoire occupancy in posttreatment samples, demonstrating that *TBX21* is a marker of clonal expansion (*n* = 17; *P* = 8.5 × 10^−7^, two-sided Wilcoxon rank sum test; boxplot as in **c**). **g**, Putative CMV-reactive effector cells have raised *TBX21* relative to non-reactive cells at baseline (*n* = 16; *P* = 1.6 × 10^−14^, two-sided Wilcoxon rank sum test; boxplot as in **c**). **h**, ATAC-seq data indicate that *TBX21* is significantly more accessible in murine CMV-reactive inflationary T cells (M38) relative to T_CM_ and naive counterparts. The region with the maximum change in accessibility between M38 compared with T_CM_ cells at the *TBX21* locus was 3.8 log_2_FC, *P*_adj_ = 3.8 × 10^−17^. **i**, *TBX21* is induced in CMV nonreactive clones in CMV^+^ individuals (*n* = 7, *P* = 4.4 × 10^−6^), while a fall is observed in seronegative patients (*n* = 8, *P* = 3.0 × 10^−8^). *P* values were derived from two-sided Wilcoxon signed-rank tests; boxplots were as in **c**. DN, double-negative T cells; GD, gamma-delta T cells; MAIT, mucosal-associated invariant T cells; TRB, T cell receptor beta chain. Source data

**Extended Data Fig. 4:**
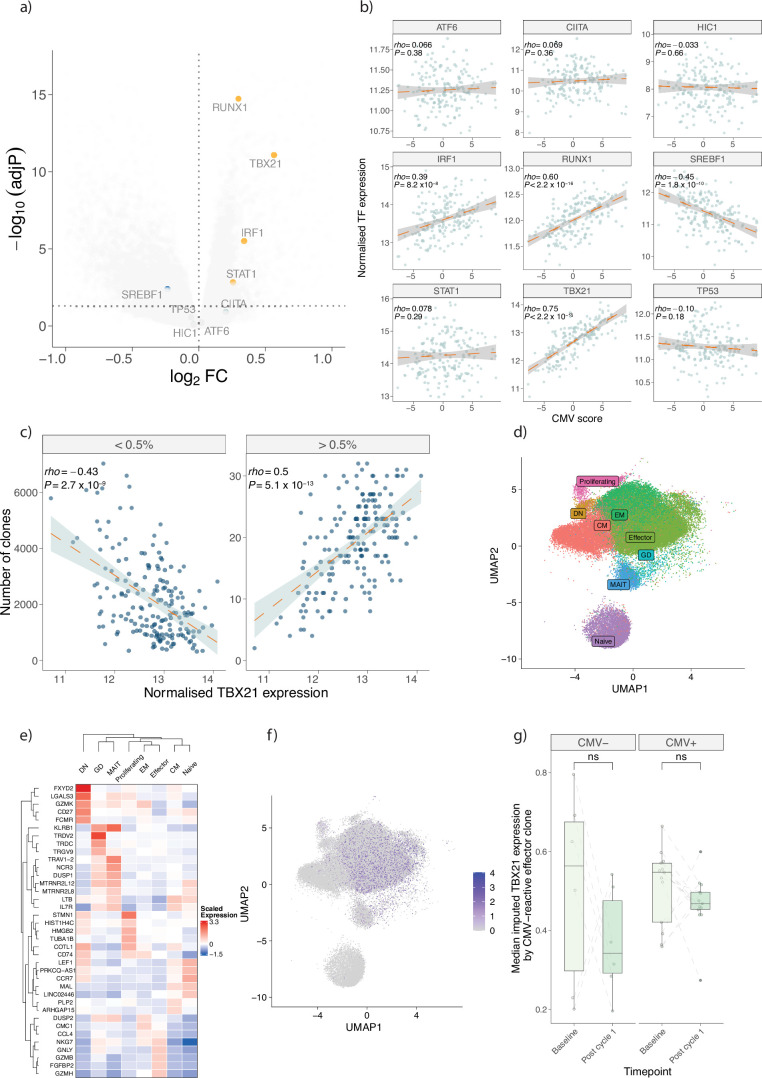
Association of TBX21 expression with clonality and cell subsets. (**a**) Differential expression of transcription factors with altered activity in CMV seropositivity in baseline CD8^+^ samples, y-axis shows Benjamini-Hochberg-corrected -log_10_(Padj.) derived from negative binomial Wald test using CMV- samples as a reference (n = 111 CMV-, n = 95 CMV+). (**b**) Correlation of transcription factors with induced activity in CMV seropositivity with CMV score (n = 181, Spearman’s Rank Correlation test, rho denotes the Spearman rho, and P value is from two-sided t-test) post cycle 1. (**c**) *TBX21* correlation with with number of CD8^+^ clones stratified by repertoire occupancy post one cycle of immunotherapy (n = 180, Spearman’s Rank Correlation test, rho and P value as defined in (**b**)). (**d**) UMAP plot of CD8^+^ T-cell subsets in single-cell RNA-seq data. (**e**) Differential expression of log-transformed cell subset marker genes in single-cell data. (**f**) Feature plot of *TBX21* expression in single-cell data. (**g**) *TBX21* expression dynamics in putative CMV-reactive effector clones in scRNA-seq data, stratified by CMV status (n = 7 CMV+: P = 0.34, n = 8 CMV-: P = 0.16; two-sided Wilcoxon signed-rank test; lower and upper box hinges represent the 25th to 75th percentiles, the central line represents the median and the whiskers extend to the highest and lowest values no greater than 1.5 × interquartile range). Source data

The conservation of the CMV-associated transcriptional profile across seropositive patients suggested it could be driven by specific TFs, as is observed in murine models of MCMV-induced memory inflation^37^. To identify these, we interrogated CMV-associated CD8^+^ T cell genes with decoupleR^38^, which infers regulatory TFs from specific gene sets. This highlighted 9 of 795 CD8^+^ T cell-expressed TFs demonstrating positive association with CMV-regulated genes after correcting for multiple testing, of which 4 were significantly upregulated in CD8^+^ T cells from CMV^+^ patients (Extended Data Fig. 4a). Correlation analysis between CMV score and expression of each CMV-associated TF demonstrated *TBX21*, encoding T-bet, to be the most strongly associated (*ρ* = 0.75, *P* < 2.2 × 10^−16^; Fig. 4a and Extended Data Fig. 4b), indicating a core role for *TBX21* in the CD8^+^ T cell response to CMV. This observation fits with the known immunological function of T-bet, a key determinant of CD8^+^ T cell effector activity^39^ that is associated with terminally differentiated anti-viral CD8^+^ T cells^40^. We subsequently explored the expression of *TBX21* across MM patient CD8^+^ T cell bulk RNA-seq data before and after ICB. Flow cytometry-based analysis of pretreatment CD8^+^ cell subsets showed a strong relationship between *TBX21* expression and subset composition per patient, with *TBX21* correlating with proportion of CD8^+^ TEMRA cells, and highly anti-correlated with naive cells (Fig. 4b). *TBX21* expression displayed a similar relationship with clone size to that observed for CMV score and, consistent with the crucial role of *TBX21* in memory inflation^37^, was correlated with increasing number of post-ICB large clones (*ρ* = 0.50, *P* = 5.1 × 10^−13^; Extended Data Fig. 4c). In keeping with *TBX21* being a driver of the CMV-associated signature in these cells, *TBX21* expression in response to ICB was similar to the CMV score—with increased baseline expression of *TBX21* in CMV^+^ individuals, while *TBX21* induction was only observed in CMV^−^ patients receiving cICB (*P* = 0.0033, Wilcoxon signed-rank test; Fig. 4c). Interestingly, sICB was associated with a slight but significant reduction in *TBX21* expression in CMV^−^ individuals (*P* = 0.0026). Similarly, although subset composition remained stable in CMV^+^ patients across treatments, CD8^+^ GZMB^+^ T cells significantly increased in cICB-treated but not sICB-treated CMV^−^ patients (Fig. 4d). Granzyme B is regulated by *TBX21* (ref. ^41^), further corroborating cICB-specific induction of cytotoxic responses in CMV^−^ patients.

To refine observations made from bulk CD8^+^ T cell RNA-seq, we performed analysis at the single-cell level, exploring the relationship between *TBX21* expression, CD8^+^ T cell subsets and individual T cell clones (Extended Data Fig. 4d–f). We generated single-cell RNA-seq (scRNA-seq) data for 18 patients with MM before and after sICB treatment (median 1,572 cells per sample baseline/1,683 cells post-cycle 1). *TBX21* was robustly expressed in CD8^+^ T effector (T_Eff_) cells, corresponding to the flow cytometry TEMRA subset, consistent with previous descriptions^39^, as well as γδ T cells, T_EM_ populations and, to a lesser extent, mucosal-associated invariant T cells (MAIT cells) (Fig. 4e). We therefore focused upon the T_Eff_ subset which had the highest median *TBX21* expression. Conserved clonal patterns of gene expression are indicative of shared antigen targets and functionality, with clonal expansion reflecting antigen recognition. We found such expanded clones (those >0.5% of total repertoire) had significantly increased *TBX21* expression after treatment (Fig. 4f), reinforcing the relationship between CMV, *TBX21* and clonal expansion. To explore *TBX21* expression with improved granularity, we used TCRMatch^42^ to identify cells carrying putative CMV-reactive TCR. These cells had elevated *TBX21* expression at baseline compared with those not carrying CMV-reactive TCR (Fig. 4g), indicating a relationship between TBX21 and CMV reactivity. To validate this observation, we explored MCMV-reactive murine T cells selected on reactivity to the MCMV epitope M38 using Assay for Transposase-Accessible Chromatin using sequencing (ATAC-seq). These cells showed open chromatin across *TBX21* that was markedly more accessible compared with central memory cells (3.8 log_2_ fold-change, *P*_adj_ = 3.8 × 10^−17^; Fig. 4h), inferring the direct relationship between CMV reactivity and *TBX21* expression. Finally, we explored the effect of ICB treatment on *TBX21* expression with reference to the putative reactivity of individual clones. We found that in CMV^+^ patients, *TBX21* is induced following sICB in clones not carrying a CMV-reactive TCR (Fig. 4i) but not in those reactive against CMV (Extended Data Fig. 4g). Such an effect was not seen, however, in seronegative patients, in keeping with a CMV-dependent bystander effect on *TBX21*, as per the bulk data. This observation indicates that, in the context of anti-PD-1 immunotherapy, CMV seropositivity has profound immuno-modulatory effects on T cells extending beyond clones specific for CMV antigens.

## CD8^+^ T cell *TBX21* expression associates with durable ICB response

**Fig. 5:**
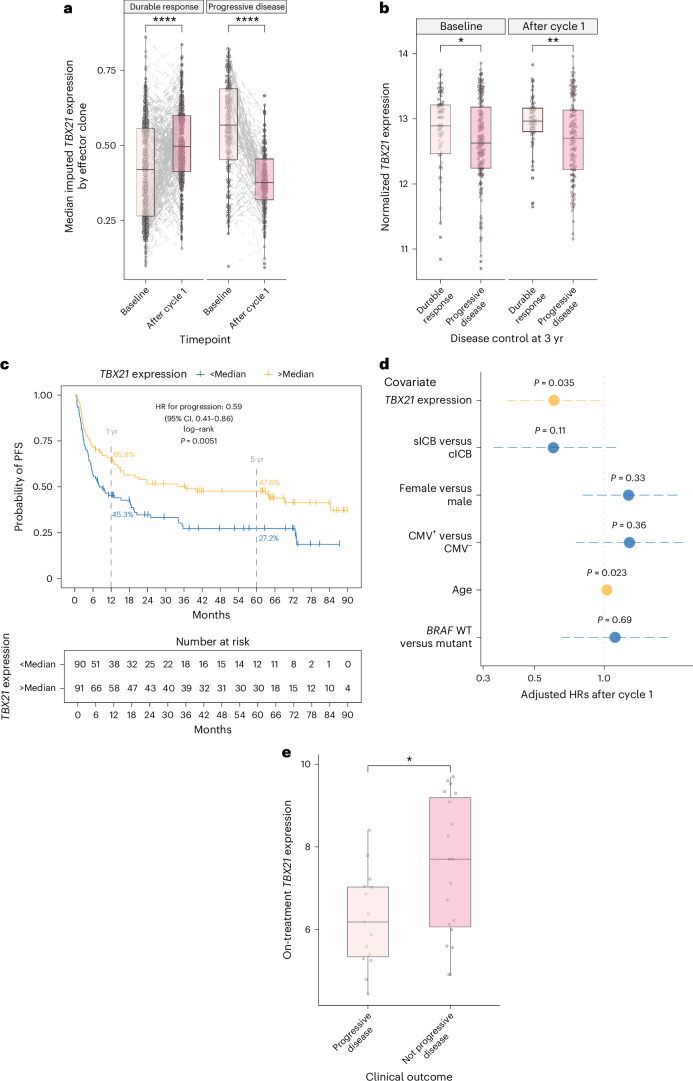
Post-ICB *TBX21* expression is associated with improved MM survival. **a**, Median imputed *TBX21* expression in effector clones from scRNA-seq data, stratified by disease control 3 yr after initiation of systemic treatment for metastatic disease, highlighting highly significant induction of *TBX21* in patients who respond to treatment (*n* = 8, *P* = 6.2 × 10^−11^) while a significant downregulation is noted in patients who progress (*n* = 7, *P* < 2.2 × 10^−16^). *P* values from two-sided Wilcoxon signed-rank test; lower and upper box hinges represent the 25th to 75th percentiles, the central line represents the median and the whiskers extend to the highest and lowest values no greater than 1.5 × IQR. **b**, Bulk CD8^+^ *TBX21* according to disease control at 3 yr after initiation of systemic treatment (baseline: *n* = 211, *P* = 0.049; post-cycle 1: *n* = 163, *P* = 0.0080; two-sided Wilcoxon rank sum test; boxplots as in **a**). **c**, Kaplan–Meier analysis demonstrates significantly improved PFS in patients with above median *TBX21* after one cycle of immunotherapy (*n* = 181, *P* = 0.0051, two-sided log-rank test). **d**, Cox proportional hazards model indicates that increased posttreatment *TBX21* is associated with improved OS when adjusting for ICB type, age, sex, *BRAF* mutation status and CMV status (HR_adj_ = 0.61; 95% CI, 0.38 to 0.96; *P* = 0.035 derived from Wald test). Increased age is associated with increased risk of death when controlling for *TBX21* (HR_adj_ = 1.03; 95% CI, 1.00 to 1.05; *P* = 0.023, Wald test). **e**, Bulk RNA-seq from tumor samples, isolated from patients on sICB treatment, indicates that *TBX21* is raised in patients with no progression compared with those who progress (*n* = 19 not progressive disease, *n* = 15 progressive disease; *P* = 0.036, two-sided Wilcoxon rank sum test; boxplots as in **a**). Source data

**Extended Data Fig. 5:**
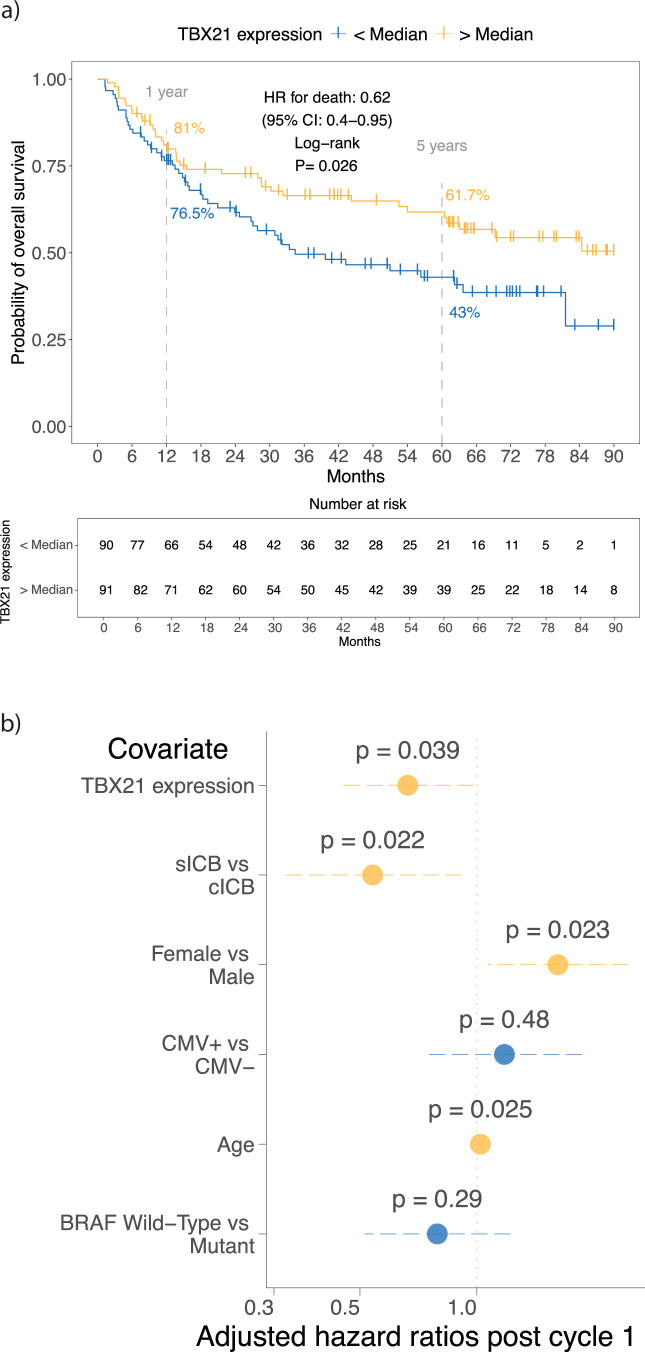
*TBX21* expression is associated with survival metrics. (**a**) Kaplan Meier analysis demonstrates significantly improved in OS in patients with above median *TBX21* post one cycle of immunotherapy (n = 181, P = 0.026, two-sided log-rank test). (**b**) Cox proportional hazards model indicates that increased post-treatment *TBX21* is associated with improved PFS when adjusting for ICB type, age, sex, *BRAF* mutational status and CMV status (n = 181, HR_adj_ = 0.66, CI 0.45:0.98, P = 0.039, Wald test). Source data

We reasoned that if the interaction between CMV status and immune response to ICB is influenced by *TBX21* expression, then CD8^+^ T cell *TBX21* expression may predict response to ICB independent of CMV. Analysis of all patients within the scRNA-seq dataset demonstrated that clones from patients with a durable response to ICB (defined as absence of progression within 3 yr of treatment initiation) displayed a pronounced induction of *TBX21* expression following treatment administration. Conversely, clones from patients with progressive disease did not show this response, instead displaying high pretreatment *TBX21* expression with significant reductions in expression upon treatment (Fig. 5a). We further assessed the relationship between *TBX21* expression and outcome from CD8^+^ T cell bulk RNA-seq data over 3-yr patient outcomes, finding baseline differences in *TBX21* expression according to outcome that became more pronounced after one cycle of ICB (Fig. 5b). We formally assessed the predictive relationship between posttreatment CD8^+^ T cell *TBX21* expression and clinical outcome using univariate Kaplan–Meier analysis across all ICB-treated patients with MM with an on-treatment sample taken at the second treatment cycle (after cycle 1, *n* = 181). This showed a significant difference in median progression-free survival (PFS) based on *TBX21* expression alone, with patients with below median expression having a median PFS of 8.0 months versus 37 months for those with above median expression (HR = 0.59; *P* = 0.0051, log-rank test; Fig. 5c), that extended to OS (HR = 0.62; *P* = 0.026, log-rank test; Extended Data Fig. 5a). Finally, we assessed the relationship between CD8^+^ T cell *TBX21* expression and survival using multivariable analysis, controlling for age, sex, ICB type, *BRAF* status and CMV status. This demonstrated that CD8^+^ T cell *TBX21* expression was the crucial predictor of both PFS (Extended Data Fig. 5b) and OS (Fig. 5d), with no other covariates showing association with outcome except increasing age which, once *TBX21* expression had been controlled for, was negatively predictive (*P* = 0.023). Finally, to validate the role of *TBX21* in determining response to ICB in the tumor, we assessed an RNA-seq dataset of biopsied melanoma samples from sICB-treated patients^43^. Consistent with a role for *TBX21*-expressing T cells in response to ICB in the periphery, we observed that *TBX21* was significantly elevated in patients without progression (*P* = 0.036, Wilcoxon rank sum test; Fig. 5e). Taken together, these findings highlight that CMV promotes T cell cytotoxicity and maturation not limited to CMV-reactive clones, underlined by increased expression of *TBX21* which is independently predictive of survival in ICB-treated MM.

## CMV seropositivity and MM epidemiology

**Fig. 6:**
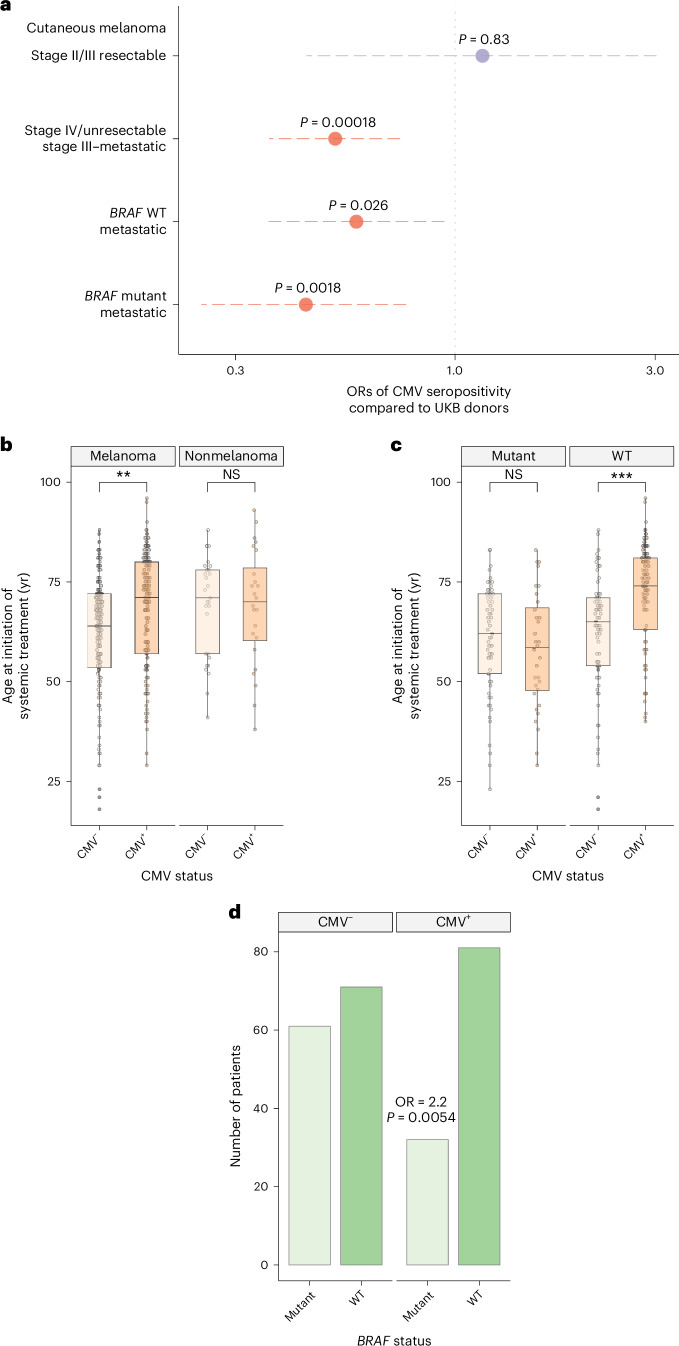
CMV interacts with MM epidemiology in a *BRAF*-mutation-dependent manner. **a**, CMV seropositivity of patients with cutaneous MM and stage II/III (age 40–70) versus those rates within UKB in white British aged 40–70 (adjuvant melanoma: *n* = 21; OR = 1.2; 95% CI, 0.44 to 3.02; *P* = 0.83; MM: *n* = 150; OR = 0.52; 95% CI, 0.36 to 0.74; *P* = 1.8 × 10^−4^; *BRAF*-WT: *n* = 82; OR = 0.58; 95% CI, 0.36 to 0.94; *P* = 0.026; BRAF mutant: *n* = 66; OR = 0.44; 95% CI, 0.25 to 0.76; *P* = 0.0018). All ORs and *P* values were obtained from a two-sided Fisher’s exact test. **b**, Presentation with cutaneous MM occurs significantly later in patients seropositive for CMV (left panel, 64 yr CMV^−^ versus 71 yr CMV^+^, *n* = 252, *P* = 0.0014), while no significant difference is noted in other ICB-treated metastatic cancer (right panel, *n* = 53, *P* = 0.89). *P* values were derived from two-sided Wilcoxon rank sum tests; lower and upper box hinges represent the 25th to 75th percentiles, the central line represents the median and the whiskers extend to the highest and lowest values no greater than 1.5 × IQR. **c**, Relationship between age of presentation with MM and *BRAF* V600 mutation status according to CMV serostatus (left panel: *BRAF* V600 mutated, right panel: *BRAF* WT). There is no significant difference in age of presentation of *BRAF*-mutated patients according to CMV serostatus (*n* = 93, *P* = 0.33), whereas a highly significant effect is noted in patients with *BRAF-*WT MM (right panel, 65 yr CMV^−^ versus 74 yr CMV^+^, *n* = 152, *P* = 1.3 × 10^−4^, two-sided Wilcoxon signed-rank test; boxplots as in **c**). **d**, Breakdown of *BRAF* mutation status versus CMV serostatus across all patients with cutaneous MM in the cohort (OR = 2.2; 95% CI, 1.2 to 3.9; *P* = 0.0054, two-sided Fisher’s exact test). Source data

In view of the interaction between CMV and ICB response, we questioned whether CMV might also act to influence MM development. Comparative analysis of CMV seropositivity within the UKB (*n* = 7,885, white British, aged 40–70) with OxCITE cohort patients in the same age group demonstrated a significantly reduced rate of CMV seropositivity in patients with cutaneous MM (MM 55 of 138, 40%; UKB 4,425 of 7,885, 56%; OR = 0.52, *P* = 1.8 × 10^−4^). Conversely, the CMV seropositivity rate in patients receiving adjuvant ICB for stage II/III resectable melanoma did not differ from the UKB, although this is a smaller sample size. The reduced CMV seroprevalence in the MM group was observed across patients with both *BRAF* mutant and wild-type (WT) disease, although the effect was accentuated in *BRAF* mutants (*BRAF*-mutated: OR = 0.44, *P* = 0.0018; *BRAF*-WT: OR = 0.58, *P* = 0.026; Fig. 6a).

**Extended Data Fig. 6:**
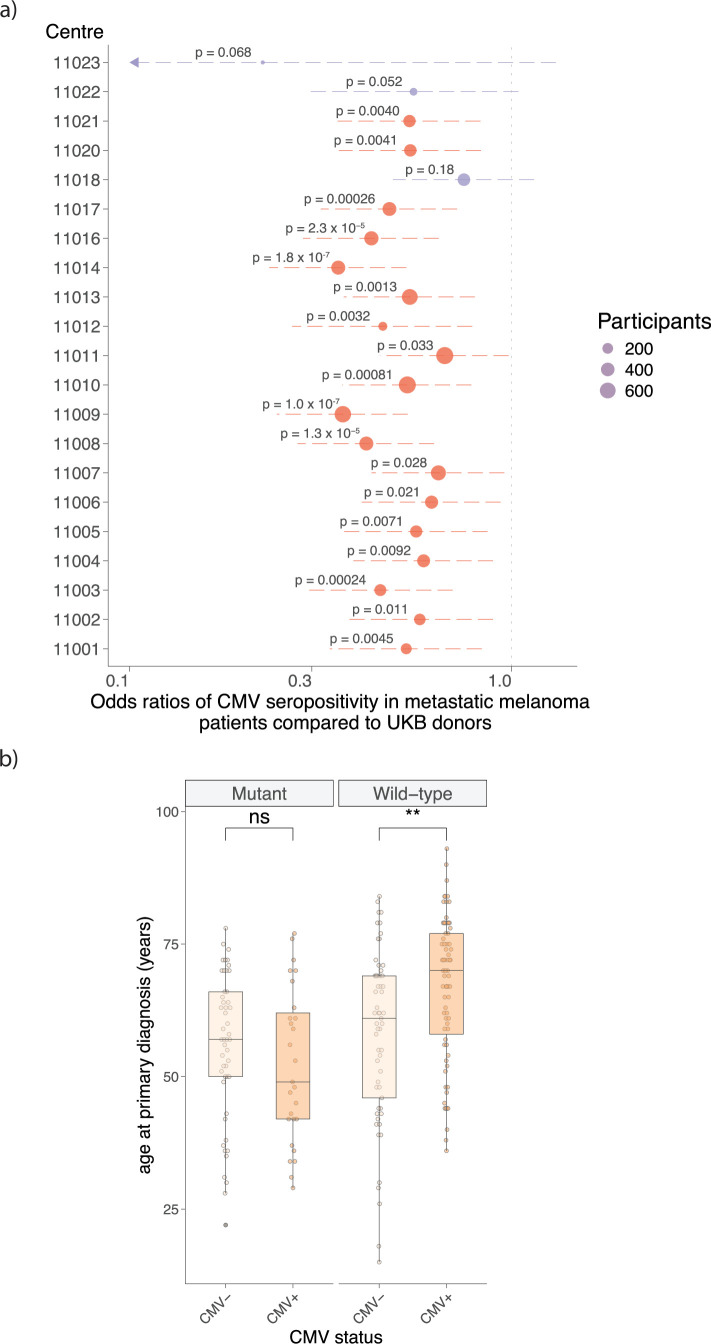
Relative CMV seropositivity across UKB recruitment centres and association with age at diagnosis of primary. (**a**) Relative depletion of CMV+ individuals in MM cohort versus UKB data according to recruitment centre (n = 150): all ORs and P values were obtained from a two-sided Fisher’s exact test. (**b**) Age of diagnosis of primary cutaneous melanoma by CMV serotype and *BRAF* status (CMV serology determined at onset of systemic treatment for metastatic disease), (n = 204, P_Mutant_ = 0.25, P_Wild-type_ = 0.0019, two-sided Wilcoxon rank sum test; lower and upper box hinges represent the 25th to 75th percentiles, the central line represents the median and the whiskers extend to the highest and lowest values no greater than 1.5 × interquartile range). Source data

A permutation analysis pairing cutaneous MM with UKB patients matched on age and sex produced a similar observation (median *P* = 0.0053, range: 0.0036–0.0078, Fisher–Pitman test), while subgroup analysis against donors from individual UKB collection centers demonstrated a consistent direction of effect, arguing against sociodemography driving the finding (Extended Data Fig. 6a). Interestingly, we found CMV^+^ patients initiating ICB for cutaneous MM were on average older (64 yr CMV^−^ versus 71 yr seropositive, mean 6.0 yr difference; 95% CI, 2.0 to 9.0; *P* = 0.0014, Wilcoxon rank sum test), an effect not seen in other metastatic ICB-treated cancers (Fig. 6b and Extended Data Table 2). Notably, however, there was no difference in the age of ICB initiation between seropositive and seronegative patients with *BRAF*-mutated melanoma (*P* = 0.33). Conversely, *BRAF*-WT CMV^+^ patients presenting with metastatic disease were a mean 9.0 yr older than seronegative counterparts (95% CI, 3.0 to 13.0 yr; *P* = 1.3 × 10^−4^, Wilcoxon rank sum test; Fig. 6c). Formal testing identified an interaction of age of presentation according to CMV seropositivity with *BRAF* mutation status (*P* = 0.0029, analysis of variance), suggesting that the described later presentation of *BRAF*-WT metastatic disease^44^ may in part be due to CMV. Indeed, no significant difference in age of treatment initiation was noted between *BRAF* mutant and WT MM when analysis was confined to CMV^−^ patients (*P* = 0.47, Wilcoxon rank sum test). Further supporting an interaction between *BRAF* mutation status and CMV, fewer CMV^+^ patients with *BRAF*-mutated MM were observed than expected (31 of 92 versus 82 of 153, *P* = 0.0054, Fisher’s exact test versus *BRAF* WT; Fig. 6d). Finally, to explore where CMV acts in MM development, we investigated the age of first presentation of primary melanoma for 204 of 245 patients who had an identified primary tumor antecedent to the development of metastatic or unresectable disease. While no difference in time from primary diagnosis to metastatic disease according to CMV status was seen, CMV seropositivity was associated with a significant difference in the age of diagnosis of primary *BRAF*-WT but not *BRAF*-mutated melanoma (*P* = 0.0019; Extended Data Fig. 6b). Here we are looking only at patients who developed metastatic disease—whether this association holds true across all primary melanoma requires further investigation.

## Discussion

We have comprehensively assessed the effect of CMV on immunological and oncological response to ICB in melanoma. We find CMV^+^ patients with MM have clinically favorable hematological markers before treatment, namely reduced NLR, fewer T_reg_ cells, increased T cell clonality and cytotoxicity, and a distinct CD8^+^ T cell transcriptomic profile, with upregulation of pathways including TCR and IFNγ-mediated signaling. This corresponds with effector skewing of CD8^+^ subsets as assessed by flow cytometry. After one cycle of ICB we find that combination anti-CTLA-4/anti-PD-1, but not anti-PD-1 alone, leads to induction of cytotoxicity and CMV-associated genes in CMV^−^ individuals, partially compensating for the transcriptomic effect of CMV. Correspondingly, OS does not differ between seropositive and seronegative cICB recipients. Conversely, CMV is associated with improved OS after sICB, an effect mirrored by increased recurrence-free survival in seropositive resected stage II/III melanoma sICB recipients. Synergism between CMV and anti-PDL1-directed immunotherapy has previously been described in murine intratumoral CMV infection studies^45^. In patients receiving anti-PD-1 and anti-LAG-3 for melanoma, enhanced responses were noted in patients with increased baseline T cell clonality linked to CMV^46^. We find *TBX21*, encoding T-bet, is associated with CMV status, with T_Eff_ cells having the highest *TBX21* expression, especially in putative CMV-reactive clones. The largest ICB-induced gain in *TBX21* expression was observed in non-CMV-reactive T_Eff_ cells, however, with this effect confined to seropositive patients—suggesting a conditional bystander effect of CMV. Crucially, posttreatment *TBX21* expression predicts OS in both univariate and multivariable analyses across all patients with MM, irrespective of CMV status, suggesting a key role for this TF in mediating both CMV immunity and ICB response. Using published intratumoral data, we also see increased *TBX21* expression after anti-PD-1 in non-progressing melanoma^43^. However, while our analysis supports *TBX21* driving the CMV effect, CMV has a pleiotropic impact on CD8^+^ T cell gene expression, including the induction of other TFs. One such example is *ZNF683*, which was recently associated with enhanced response to anti-PD1 therapy in B cell lymphoma^26^. Thus, it is likely that other TFs that promote CMV-associated responses will contribute to these findings. Of translational relevance, we find that CD8^+^ T cells from CMV^−^ patients gain a CMV^+^-like transcriptional profile following treatment with cICB, but not sICB. Elucidating the mechanisms whereby anti-CTLA-4 induces this signature in seronegative individuals requires further in-depth studies, but it is noteworthy that T_reg_ cells, a known target of anti-CTLA-4-mediated antibody-dependent cellular cytotoxicity, are significantly raised in CMV^−^ individuals, and thus this difference in number coupled to the absence of anti-CTLA-4 in sICB is a plausible cause of divergent clinical outcomes. These findings have implications for clinical practice; treatment with cICB has an improved OS benefit compared with sICB at the expense of markedly elevated rates of high-grade irAEs^47^. Furthermore, a substantial proportion of patients gain long-term disease control with sICB alone and the identification of these patients is of high clinical importance. Thus, CMV serostatus may allow personalization and stratification of these treatments.

Our analysis has also revealed a clinically meaningful association between CMV and ICB-induced irAEs. These have previously been associated with increased TCR diversity which corresponds to T cell naivety^48^. As such, CMV-associated reduction in naive and central memory cells would be anticipated to be intrinsically protective. Specifically, we find CMV infection bestows reduced risk of grade 3+ irAEs, in particular colitis and pneumonitis. These organs are frequent sites of CMV reactivation in immunocompromised individuals^49–51^. Given CMV modulates tissue immunity^52,53^ and resident memory T cells are associated with ICB-induced colitis^54^, we postulate that CMV alters the sensitivity of resident immune cell subsets to ICB. In keeping with divergent effects of CMV across organs, seropositive patients display increased rates of prognostically favorable cutaneous irAEs^31,35^. Interestingly, in an analysis linking Epstein–Barr virus infection to development of multiple sclerosis, CMV infection was found to be protective against multiple sclerosis development^55^, replicating earlier findings^56^. Thus, it is increasingly apparent that different CVIs can have pleiotropic organ-specific effects on inflammatory and autoimmune conditions. Dissection of these effects will require immunophenotyping of the relevant tissues, taking into account CMV serostatus, and, while outside the scope of this original report, is of high importance to improved understanding of ICB toxicity.

Finally, we detected reduced CMV seroprevalence in patients with MM compared with the UKB—a large, demographically relevant population control cohort. This observation is in keeping with the chronic anti-viral response influencing anti-tumor immunity, with CMV conferring protection against MM onset. Interestingly, the protective effect of CMV is conditional upon *BRAF* mutation status, with significantly fewer *BRAF*-mutated CMV^+^ patients with MM observed than expected, whereas *BRAF*-WT MM presented later in CMV^+^ individuals. The apparent increased sensitivity of *BRAF*-mutated tumors to a CMV-related preventative effect is consistent with *BRAF*-mutated melanomas invoking an immune response distinct from that of *BRAF*-WT tumors, with reduced CD8^+^ T cell infiltration^57^ and improved outcomes to cICB^47^. Our observations add to work exploring associations between CMV and cancer mortality, with either deleterious or no effects noted in pan-cancer analyses^58–60^, while a protective effect has been described in some cancer-specific analyses^61^. Given the observed interaction with *BRAF* status, we speculate CMV and other CVIs may influence the development of other cancers in a more nuanced, cancer-specific and potentially mutation-dependent, manner.

While this is one of the largest analysis of interactions between CMV status and ICB response and toxicity in a prospectively recruited patient cohort to date, it is not without limitations. Primarily, these data are observational and, while work is in progress to generate a functional model, caution must be applied when inferring causation. Furthermore, while we detect evidence of replication of CMV protection against toxicity in non-melanoma OxCITE patients, the numbers of non-melanoma samples are small and future studies regarding interactions between CMV and response and toxicity to ICB in non-melanoma cancers are vital. Although we did not find CMV to be associated with age of diagnosis of non-melanoma metastatic cancer, this analysis was of multiple different tumor types and larger, cancer-type-specific prospective studies are required. While we cannot exclude patient willingness to participate in research as a potential source of self-selection bias, we do not expect this to have had any significant effect on results. We did not have objective response data available for this cohort, meaning we could not assess the impact of CMV serostatus on outcome measures such as best overall response or objective response rate. However, given these measures are typically used as surrogate outcomes for the more clinically relevant OS, we believe our data to be robust. Finally, exploring the relationship between CMV and MM in a country with dissimilar CMV seroprevalence to the United Kingdom would be ideal. This is complicated by countries with the highest incidence of MM having similar CMV seroprevalence^62–64^. Intriguingly, melanoma *BRAF* mutation incidence shows striking variation across regions with similar ultraviolet exposure^65^, and is lower in populations with high CMV seroprevalence^66,67^. Nonetheless, given differences in other environmental factors and genetic ancestry, studies taking into account CMV status in conjunction with confounding factors are vital. Notwithstanding these points, our findings are based on a prospectively recruited, large cohort of patients receiving ICB within a standard-of-care setting, in conjunction with relevant population controls and public tumor data. For each patient we generate a multi-dimensional dataset including annotated follow-up, CMV serotyping and T cell transcriptional profiling, and, for a subset of patients, scRNA-seq and flow cytometry analysis. Given the relatively balanced seroprevalence of CMV in the United Kingdom, the cohort is powered to detect clinically meaningful differences with high confidence and to control for confounding factors.

This work has ramifications for our immunological understanding and future clinical applications. Firstly, our data further challenge the dogma that CMV infection has solely deleterious effects on immunity^27^. We find that CMV polarization of T cell immunity towards an effector phenotype is associated with improved immune surveillance and tumor rejection. Our data also show that CMV buffers the effect of ageing in MM, with age only associated with an increased hazard for death once CMV serostatus has been controlled for. Secondly, this study has important clinical implications as it suggests that CMV^−^ patients are at risk of poorer outcomes if not treated with cICB, yet are also at increased risk of high-grade irAEs. CMV^−^ patients may therefore particularly benefit from irAE mitigation strategies. While CMV may similarly interact with the response of other tumor types to ICB, the previous largest reported study in lung cancer did not find a direct association of seropositivity with OS^68^, and further studies of the effect of CMV and other CVIs on the responses of other immunosensitive cancers to ICB are required. Finally, as immunotherapies become increasingly complex and costly, we show that nonspecific immune stimulation from CVI may have substantial epidemiological and clinical impacts in melanoma. The identification of *TBX21*, encoding T-bet, as a core TF associated with the benefit of CMV seropositivity delineates a molecular pathway that could be modulated to enhance ICB efficacy. Our findings also have implications for trial design of cancer vaccines where it will be important to dissect benefits brought by targeted agents from those conferred by generic adjuvants. As such, this work reinforces the importance of tumor-extrinsic factors that influence systemic immunity in the development of metastatic cancer and response to immunotherapy.

## Methods

### Ethics approval and consent to participate

Informed consent was given by all patients to donate samples to the Oxford Radcliffe Biobank (Oxford Centre for Histopathology Research ethical approval reference 19/SC/0173, project nos. 16/A019, 18/A064 and 19/A114) and grant access to their routine clinical data. There was no compensation for this consent.

### Patients

Recruitment occurred between 23 November 2015 and 8 July 2024. Patients eligible for ICB in the Oxford University Hospitals NHS Foundation Trust were recruited prospectively. These included patients with advanced or metastatic melanoma, renal cell carcinoma, mismatch repair-deficient colorectal cancer, mesothelioma and cutaneous squamous cell carcinoma. Patients due to receive adjuvant ICB for melanoma and renal cell carcinoma were also eligible. Immunological and clinical outcome analyses were confined to patients receiving treatment for melanoma who were treated with either cICB (ipilimumab 3 mg kg^−1^ every three weeks (q3w) plus nivolumab 1 mg kg^−1^ q3w for ≤4 cycles followed by maintenance nivolumab) or sICB (pembrolizumab 200 mg q3w or 400 mg every six weeks (q6w), or nivolumab 240 mg every two weeks (q2w) or 480 mg every four weeks (q4w)).

Cohorts incorporated for each figure are listed in Supplementary Table 7.

(OxCITE melanoma cohort, *n* = 341, median follow-up 50.3 months; patients with MM median follow-up: 58.2 months, adjuvant melanoma median follow-up: 24.5 months.)

### Mice

C57BL/6 mice were obtained from Harlan, and intravenously infected with 1 × 10^6^ plaque-forming units MCMV (Strain Smith; ATCC: VR194). Splenocytes were isolated 85 d after infection. Experiments were performed according to UK Home Office regulations (project license number PPL 30/2235 and 30/2744). CD8^+^ T cells were isolated from splenocytes by negative selection (Miltenyi Biotec) and sorted (MoFlo; Beckman Coulter) as: M38-tetramer^+^CD44^+^CD62L^−^ (‘M38’, inflationary), M38^−^CD44^+^CD62L^+^ (‘CM’, central memory) and M38^−^CD44^−^CD62L^+^ (‘Naive’, naive).

### Clinical outcomes

Patient demographic, clinical, hematological, biochemical and radiological data were extracted from electronic patient records. All patients had at least 3 months of follow-up and complete toxicity data available. Survival analysis was restricted to patients with at least 6 months of follow-up. Staging was recorded according to American Joint Committee on Cancer (AJCC) 8th edition criteria, with Tumour Node Metastasis (TNM) 8th edition criteria for Metastasis M1a–d classification^69,70^. Hematological and biochemical data were primarily confined to those within the Oxford University Hospitals Trust catchment area. Calculation of lymphocyte count and NLR was based on the last full blood count taken before the first cycle of ICB (median 5 d before treatment, interquartile range (IQR): 14–2 d). Baseline serum lactate dehydrogenase was dichotomized into above and below the upper limit of normal according to Oxford University Hospitals pathology criteria. All patients who received at least one cycle of ICB were included in the analysis. irAEs were assessed according to the National Cancer Institute’s Common Terminology Criteria for Adverse Events, version 5.0. PFS was determined as time from treatment initiation to clinical or radiological evidence of progression or death. Objective response was not available for this cohort. OS was defined as time from treatment initiation to death from any cause.

### Sample collection

Up to 50 ml of whole blood was collected from each patient, in EDTA vacutainer tubes, immediately before ICB administration. For samples post-cycle 1 this was at the time of receipt of 2nd cycle of ICB according to standard-of-care protocol—for ipilimumab + nivolumab (cICB) this was day 22, for sICB (pembrolizumab/nivolumab) day 22 or day 29. Plasma and PBMCs were separated immediately by density centrifugation using Ficoll-Paque. Cell-subset sorting for bulk RNA-seq and scRNA-seq was performed using Miltenyi Biotec magnetic separation, with positive selection for CD8^+^ T cells performed per manufacturer instructions at 4 °C. Baseline full blood count data were obtained from electronic patient records, generated via Sysmex XN series analyzers. The blood sample taken closest to initiation of ICB, between day −30 and day 0, was used for analysis. CMV IgG antibody titers were measured using patient plasma diluted 1:2 in HBSS from day 0 samples on an Abbott Architect i2000 following good laboratory practice within the John Radcliffe Hospital, Oxford. Median value for CMV^−^ patients = 0.2 a.u. ml^−1^ (mean 0.3 a.u. ml^−1^, IQR: 0.1–0.3 a.u. ml^−1^). For CMV^+^ patients saturating values (where a.u. > 250 a.u. ml^−1^) were read as 250 a.u. ml^−1^, median value = 87.5 a.u. ml^−1^ (mean 105.9 a.u. ml^−1^, IQR: 53–156.8 a.u. ml^−1^).

### Flow cytometry

Cryopreserved patient PBMC samples were thawed at 37 °C and washed with HBSS. Then, 1 × 10^6^ PBMCs were plated, and viability staining was performed with Near IR Fixable Viability Stain (ThermoFisher, cat. no. L34975) or Fixability Viability Stain 440UV (BD Biosciences) for 30 min at 4 °C. PBMCs were washed with HBSS supplemented with 5% FCS and BD Horizon Brilliant Stain Buffer (BD Biosciences, 563794). Staining for T cell surface markers was performed for 30 min at 4 °C using antibodies directed against markers listed in Supplementary Table 5. PBMCs were washed once in cell staining buffer (BioLegend, cat. no. 420201) and incubated with Streptavidin (BV785, BioLegend cat. no. 405249) for 30 min at 4 °C for IgG4 staining. Following surface staining, PBMCs were permeabilized with Foxp3/Transcription Factor Staining Buffer Set (Invitrogen, cat. no. 00-5523-00) for intra-nuclear staining or Transcription Factor Buffer Set (BD Biosciences, cat. no. 562574) for cytoplasmic staining, for 30 min, and incubated with intracellular staining antibodies listed in Supplementary Table 5. PBMCs were resuspended in 2% paraformaldehyde and acquisition was performed on a FACSSymphony A5 Cell Analyzer (BD Biosciences) or a Fortessa X-20. Data were analyzed using FlowJo v.10.7.1 (BD Biosciences).

### Nucleic acid extraction

Postselection cells were spun down at 4 °C and resuspended in 350 µl of RLTplusbuffer with 1% DTT and transferred to 1.5-ml Eppendorf tubes. Samples were stored at −80 °C for batched RNA extraction. Homogenization of the sample was carried out using the QIAshredder kit (Qiagen). The AllPrep DNA/RNA/miRNA Kit (Qiagen) was used for RNA extraction. DNase I was used during the extraction protocol to minimize DNA contamination. RNA was eluted into 35 µl of RNase-free water. The amount of RNA present was quantified by Qubit analysis, and RNA samples were stored at −80 °C until sequencing.

### Bulk RNA-seq preparation and analysis

RNA was thawed on ice before messenger RNA isolation using the NEBNext Poly(A) mRNA Magnetic Isolation Module Kits. Poly(A) RNA was either sequenced using 150-base pair-end sequencing on an Illumina NovaSeq or using 75-base pair-end sequencing on an Illumina HiSeq-4000, both at the Oxford Genome Centre. Reads were aligned to CRGh38/hg38 using HISAT2 and read count data were generated using HTSeq. High-mapping-quality reads were selected based on MAPQ score using bamtools. Marking and removal of duplicate reads were performed using picard (v.1.105) and samtools was used to pass through the mapped reads and calculate statistics. A total of 236 (206 with CMV serology, 95 CMV^+^ and 111 CMV^−^) high-quality baseline CD8^+^ T cell transcriptomes were included in the analysis along with 181 (162 with CMV serology, 76 CMV^+^ and 86 CMV^−^) post-cycle 1 transcriptomes, with 170 (151 with CMV serology, 72 CMV^+^ and 79 CMV^−^) paired samples. Normalized counts were produced using DESeq2 (v.1.40.1).

DESeq2 was used to perform differential expression analysis at baseline to identify genes modulated by CMV. Age, sex and sequencing batch were corrected for in the design formula and only genes with a mean count > 10 were included in the analysis. Up- and downregulated genes were treated separately for pathway analysis using the R package XGR (v.1.1.9). The Gene Ontology Biological Process database was used with all annotated genes (*n* = 14,614) serving as a background. The ontology algorithm was specified as ‘elim’ along with an elimination *P* value of 0.01, and a hypergeometric test was employed.

CMV score was determined using the normalized counts of differentially expressed genes at baseline between CMV^+^ and seronegative patients. Gene counts were isolated for baseline and post-cycle 1 samples and were incorporated in a principal component analysis (PCA) using the R package stats. The first principal component was extracted and was used in a receiver operating characteristic (ROC) analysis (using the R package pROC v.1.18.2) to predict CMV serostatus. The number of genes to be included in the CMV score was determined through iterative PCA and ROC analyses between an adjusted *P* value threshold, obtained from the DESeq2 results, of 0.05–10^−15^ (encompassing 7,576 to 53 genes). The threshold yielding the maximum area under the curve (0.90) was selected, including 75 genes. Cytotoxicity score was determined using a set of 50 genes that had been previously found to correlate significantly with *IFNG* expression (Supplementary Table 3). The normalized counts of this gene set were extracted from baseline and post-cycle 1 samples and the score was calculated using a geometric mean.

TF activity was determined in R using decoupleR (v.2.6.0). The network from OmniPath, obtained for ‘human’ and without splitting complexes, was filtered to contain TFs that were detected in the CD8^+^ T cell count matrix, yielding 795 testable TFs. Counts data were normalized using variance stabilizing transform in DESeq2. Normalized counts data along with differentially expressed genes (false discovery rate (FDR) < 0.05) according to CMV serostatus at baseline were used in a univariate linear model for TF activity inference. TFs with induced activity in CMV seropositivity following FDR adjustment (*P*_adj_ < 0.05) were included in downstream analysis. *TBX21* normalized expression was extracted using DESeq2 for both OxCITE and Riaz datasets.

### Bulk adaptive receptor analysis

MiXCR was used to map bulk CD8^+^ RNA-seq reads on reference sequences of V, D and J genes, and to quantitate TCR clonotypes using complementarity-determining region 3 (CDR3) gene regions. The nondefault partial alignments option (OallowPartialAlignments = true) was applied to preserve partial alignments for the assembly step. Three iterations of read assembly were performed to increase the number of assembled reads containing the CDR3 region using assemblePartial action. Position quality scores were used to switch on the frequency-based correction of clonotype assembling and clustering (ObadQualityThreshold = 15). Clones were called according to the nucleotide sequence and a total of 272,886 *TRA* chains (median per sample = 1,363 chains, range = 203–4,541 chains) and 396,292 *TRB* chains (median per sample = 1,750 chains, range = 339–7,041 chains) were identified after cycle 1. Clone size was determined for *TRB* chains, with large clones being defined as those with a peripheral repertoire occupancy (determined by cloneCount/Total *TRB* count) >0.5% while small clones were those found to occupy <0.5% of the repertoire.

### scRNA-seq preparation and analysis

A combination of fresh (8 samples) and thawed (28 samples) positively selected CD8^+^ T cells were counted and oil-partitioned into single-cell droplets, followed by cell lysis and reverse transcription on a 10X Genomics Chromium System. A total of 6,000 cells were loaded onto each partitioning reaction. 5′ GEX and V(D)J libraries were constructed from complementary DNA using the Chromium Next GEM Single Cell 5′ Reagent Kit v2 (Dual Index). Droplet generation and reverse transcription were performed on individual samples, but library generation and sequencing were performed in batches. Sequencing was performed on an Illumina HiSeq-4000: 75-base pair (bp) paired-end reads for 5′ RNA libraries, 150-bp paired-end reads for the V(D)J libraries to a depth of 20,000–50,000 reads per cell. FASTQ files were generated from raw Illumina BCL outputs using Cellranger (v.6.0.1) for GEX libraries and Cellranger VDJ for V(D)J libraries. For quality control, the R package scater was used to identify single-cell outliers and the R package scran was used to identify doublets. After preprocessing, CD8^+^ T cells were filtered for expression of *CD3D* and either *CD8A* or *CD8B* along with absence of *CD14* expression. Seurat was used for further quality control, read normalization and identification of variable features. Cells used in analysis were those with >500 total unique molecular identifiers, >300 features and mitochondrial gene percentage < 20%. Genes expressed in <5 cells were also excluded. PCA was performed on scaled data, with the top 16 principal components being selected for dimensionality reduction and uniform manifold approximation and projection clustering. Cell clusters were generated between resolutions of 0.1 and 2.0 using the R package clustree, with values of 1.5–2.0 demonstrating high cluster stability. Cells were assigned to subsets based on curated transcripts per million normalized counts for CD8^+^ T cells from previous public cancer scRNA-seq datasets using SingleR^70–74^.

*TBX21* expression was imputed using the MAGIC package (v.2.0.3.999)^75^. Subset expression of *TBX21* was plotted using the ggridges (v.0.5.4) package, and the effector subset was found to have the highest median imputed expression (0.39 units), and thus downstream analysis was restricted to this subset. Clones were defined as those with a unique detectable CDR3 *TRA* and/or *TRB*. Median imputed *TBX21* was determined per effector clone for 18 sICB-treated patients at baseline (*n* = 17) and post-cycle 1 (*n* = 16) (15 paired samples). There were 1,431 (median per sample = 76 clones) clones from baseline samples and 1,557 clones from post-cycle 1 samples (median per sample = 66 clones). Clone size was determined using the same criteria as for MiXCR data.

TCRMatch was used to score *TRB* chains in the single-cell data based on putative antigen specificity. The highest-scoring antigen was assigned to each clone, and clones with a score >0.9 for CMV epitopes were assigned as CMV reactive.

### ATAC-seq

ATAC-seq was performed as described previously^76^. ATAC-seq reads from three biological replicates for each sample were mapped to the mouse genome (mm10 assembly) using Bowtie 2 (ref. ^77^). In all cases, duplicate reads were removed using Picard (markDuplicates; http://broadinstitute.github.io/picard/) and customized Python scripts were used to calculate the fragment length of each pair of uniquely mapped paired-end reads. Only one mapped read to each unique region of the genome that was less than 175 bp was kept for peak calling. Regions of open chromatin were identified by MACS (v.1.4.2)^78^ using a *P* value threshold of 1 × 10^−5^. Only regions called in all replicates were used in downstream analysis. Peak intensities were normalized as reads per kilobase million. Downstream analyses were performed with R. DESeq2 was used to identify the differential abundance of cut sites between the peaks and to perform all pairwise comparisons of the three conditions. Cut sites between peaks with an FDR < 0.05 were considered differential. Peaks were visualized using Integrative Genomics Viewer^79^.

### Statistical analysis

Statistical analyses were performed using R v.4.4.0. Survival analyses were performed using the survival (v.3.7-0) and survminer (v.0.4.9) packages, while cumulative incidence and competing risks regression used tidycmprsk (v.1.0.0). Plots were generated using ggplot2 (v.3.5.1). Unless stated otherwise, all Cox proportional hazards, cumulative risk and logistic regression analyses were performed adjusting for age, sex, *BRAF* status and single versus combination ICB. Median follow-up time was calculated using the reverse Kaplan–Meier method described by Schemper and Smith^80^. For survival analysis, patients with a minimum of 6 months of follow-up were included. Survival analysis was censored at 90 months after initiation of systemic treatment for MM. CMV effect on OS was assessed separately for patients with MM receiving sICB or cICB, initially via Kaplan–Meier curves with univariate log-rank tests then via multivariable Cox proportional hazards models for sICB. Univariate cumulative incidence estimates were calculated for time to irAEs using a semi-competing risks model, with death as the competing risk. Times to first irAE of grade 1–2 irAEs and first grade 3+ irAEs were assessed separately for their association with CMV status. Significance testing was performed using Gray’s test^81^. Competing risk regression was then used to calculate the Fine–Gray subdistribution hazard for association of CMV status and each irAE measure^82^. Adjusted ORs for irAEs were calculated using binomial logistic regression, correcting for ICB type, age, sex, *BRAF* mutation status and treatment intent. The impact of irAEs on OS was restricted to patients with MM, and was assessed using a time-dependent Cox-regression model to account for guarantee–time bias^83^. First, grade 1–2 and grade 3+ irAEs were considered as time-dependent covariates. Second, organ-specific irAEs were assessed, again as time-dependent covariates, adjusting for each different irAE and the same covariates used in the binary logistic regression analysis.

Correlation analysis was performed using a Spearman’s rank correlation test and distributions were compared using either a Wilcoxon signed-rank test, for paired data, or a Wilcoxon rank sum test, for unpaired data. ORs were generated using a Fisher’s exact test, while adjusted ORs were generated using a generalized linear model (family = ‘binomial’). Age and sex matching between the OxCITE cohort and the UKB was performed using the R package MatchIt (v.4.5.5). Permutation testing was performed using a two-tailed alternative hypothesis and 10,000 Monte-Carlo replicates, using the coin package (v.1.4-2). The matching process was bootstrapped 1,000 times and the median *P* value was determined. For all data, *P < 0.05, **P < 0.01, ***P < 0.001, ****P < 0.0001.

### Reporting summary

Further information on research design is available in the Nature Portfolio Reporting Summary linked to this article.

## Online content

Any methods, additional references, Nature Portfolio reporting summaries, source data, extended data, supplementary information, acknowledgements, peer review information; details of author contributions and competing interests; and statements of data and code availability are available at 10.1038/s41591-025-03647-1.

## Supplementary information

Supplementary Information Supplementary Fig. 1. Flow diagrams. (**a**) Epidemiology flow diagram. (**b**) Survival flow diagram. (**c**) irAE flow diagram. Supplementary Fig. 2. Flow gating strategy. Supplementary Fig. 3. Cohort matching tables. (**a**) OxCITE melanoma cohort. (**b**) OxCITE nonmelanoma cohort. (**c**) cICB-treated MM cohort with >6-month follow-up. (**d**) sICB-treated MM cohort with >6-month follow-up. (**e**) Adjuvant-treated melanoma cohort with >6-month follow-up.

Reporting Summary

Supplementary Tables 1–7 Supplementary Table 1. Differentially expressed CD8^+^ T cell genes according to CMV serostatus. Supplementary Table 2. GOBP pathway analysis of CD8^+^ T cell CMV differentially expressed genes. Supplementary Table 3. Genes incorporated into cytotoxicity score. Supplementary Table 4. CMV-associated transcription factors—output from decoupleR. Supplementary Table 5. Antibodies utilized in flow cytometry. Supplementary Table 6. Cohort missingness table. Supplementary Table 7. Figures overview.

## Source data

Source Data Fig. 1 Fig. 1a. Pre-treatment lymphocyte count. Fig. 1b. Pre-treatment neutrophil to lymphocyte ratio. Fig. 1c. CD4^+^ T cell flow cytometry. Fig. 1d. CD8^+^ T cell flow cytometry. Fig. 1e. T_reg_ flow cytometry. Fig. 1f. Bulk CD8^+^ RNA-seq data. Fig. 1g. Bulk CD8^+^ RNA-seq data.

Source Data Fig. 2 Fig. 2a. Bulk CD8^+^ RNA-seq data. Fig. 2b. Bulk CD8^+^ RNA-seq data. Fig. 2c. Overall survival data. Fig. 2d. Overall survival data. Fig. 2e. Overall survival data. Fig. 2f. Progression-free survival data.

Source Data Fig. 3 Fig. 3a. Time to toxicity data. Fig. 3b. Time to toxicity data. Fig. 3c. Toxicity management data. Fig. 3d. Toxicity site data. Fig. 3e. Colitis data.

Source Data Fig. 4 Fig. 4a. Bulk CD8^+^ RNA-seq data. Fig. 4b. Bulk CD8^+^ RNA-seq data & CD8^+^ T cell flow cytometry. Fig. 4c. Bulk CD8^+^ RNA-seq data. Fig. 4d. GZMB^+^ CD8^+^ flow cytometry. Fig. 4e. CD8^+^ scRNA-seq data. Fig. 4f. CD8^+^ scRNA-seq and scTCR-seq data. Fig. 4g. CD8^+^ scRNA-seq and scTCR-seq data. Fig. 4h. ATAC-seq data. Fig. 4i. CD8^+^ scRNA-seq and scTCR-seq data.

Source Data Fig. 5 Fig. 5a. CD8^+^ scRNA-seq data. Fig. 5b. Bulk CD8^+^ RNA-seq data. Fig. 5c. Progression-free survival data and bulk CD8^+^ RNA-seq data. Fig. 5d. Overall survival data and bulk CD8^+^ RNA-seq data.

Source Data Fig. 6 Fig. 6a. Clinical data and UKB data. Fig. 6b. Clinical data. Fig. 6c. BRAF data. Fig. 6d. BRAF data.

Source Data Extended Data Fig. 1 Extended Data Fig. 1. Pre-treatment neutrophil to lymphocyte ratio.

Source Data Extended Data Fig. 2 Extended Data Fig. 2a. Bulk CD8^+^ RNA-seq data. Extended Data Fig. 2b. Bulk CD8^+^ RNA-seq data. Extended Data Fig. 2c. Bulk CD8^+^ RNA-seq data. Extended Data Fig. 2d. Bulk CD8^+^ RNA-seq data.

Source Data Extended Data Fig. 3 Extended Data Fig. 3a. Overall survival and toxicity data. Extended Data Fig. 3b. Time to toxicity data. Extended Data Fig. 3c. Time to toxicity data.

Source Data Extended Data Fig. 4 Extended Data Fig. 4a. Bulk CD8^+^ RNA-seq data. Extended Data Fig. 4b. Bulk CD8^+^ RNA-seq data. Extended Data Fig. 4c. Bulk CD8^+^ RNA-seq data. Extended Data Fig. 4d. CD8^+^ scRNA-seq data. Extended Data Fig. 4e. CD8^+^ scRNA-seq data. Extended Data Fig. 4f. CD8^+^ scRNA-seq data. Extended Data Fig. 4g. CD8^+^ scRNA-seq and scTCR-seq data.

Source Data Extended Data Fig. 5 Extended Data Fig. 5a. Overall survival data and bulk CD8^+^ RNA-seq data. Extended Data Fig. 5b. Progression-free survival data and bulk CD8^+^ RNA-seq data.

Source Data Extended Data Fig. 6 Extended Data Fig. 6a. Clinical data and UKB data. Extended Data Fig. 6b. BRAF data.

Source Data Extended Data Table 1 Extended Data Table 1. Clinical data.

Source Data Extended Data Table 2 Extended Data Table 2. Clinical data for nonmelanoma cohort.

## Figures and Tables

**Extended Data Table 1:**
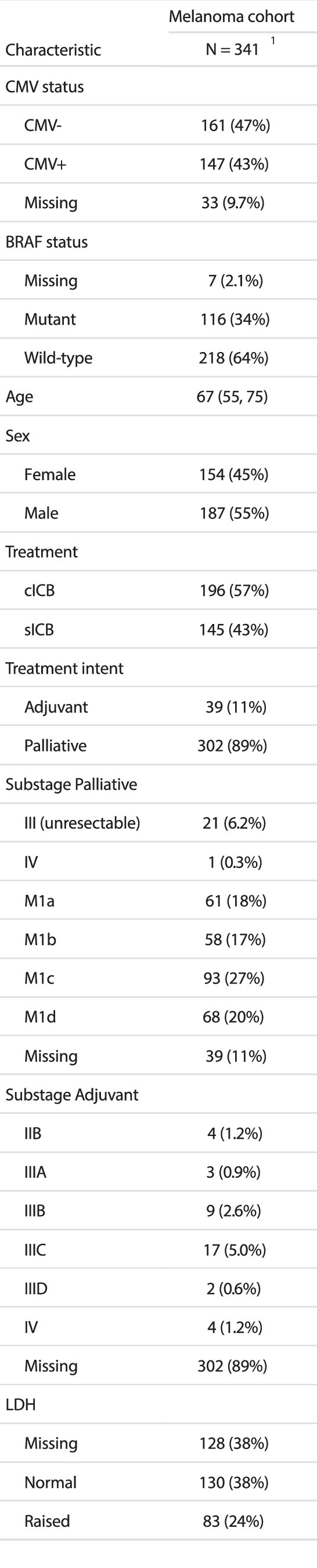
OxCITE Melanoma cohort breakdown

Source data

**Extended Data Table 2:**
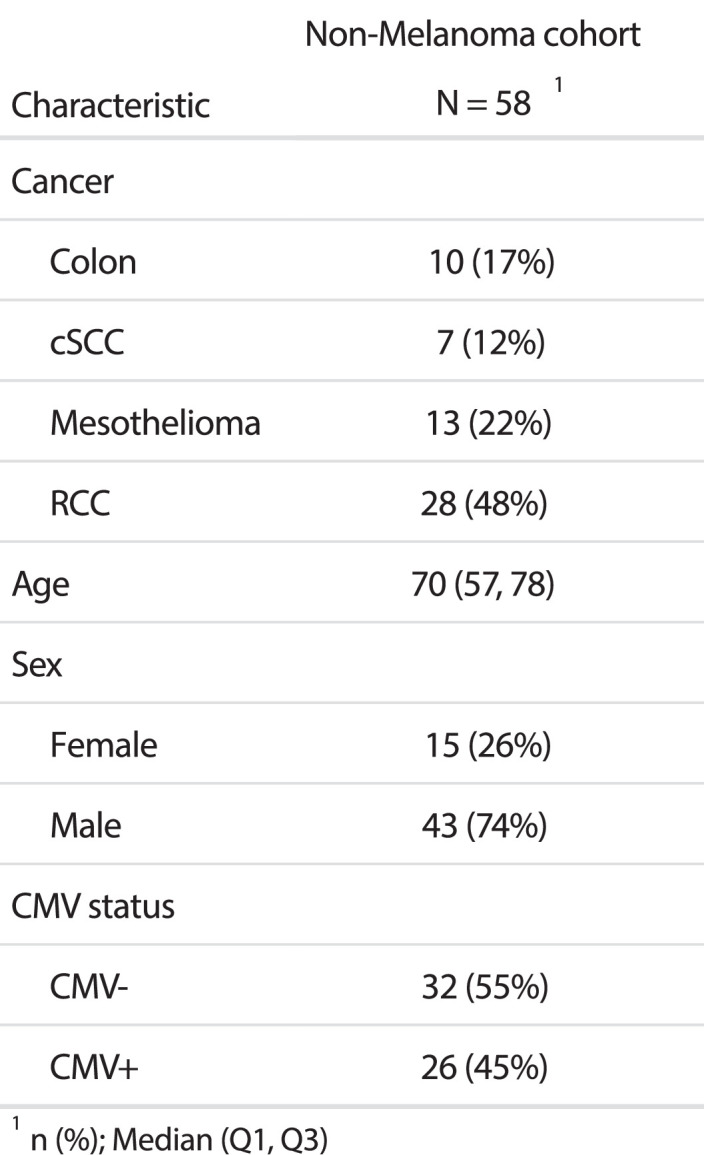
OxCITE Non-Melanoma cohort breakdown

Source data

## Data Availability

Raw sequencing data at the individual level corresponding to anonymized patients will be made available for download from EGA (EGAD00001007942) via a Data Access Arrangement between applicants and the University of Oxford 6 months from the date of publication—these data will be available for a minimum of 2 years. Requests should be addressed to benjamin.fairfax@oncology.ox.ac.uk and will be processed within the university but should take *<*6 weeks to action. Normalized gene expression matrices with associated minimal anonymized patient information (required to perform analyses to recreate the figures in the paper) is available via Oxford Research Archive (10.5287/ora-5jy9jpmaq). Source data are provided with this paper.

## Code availability

Code to perform all analyses and make all figures within the paper is provided on Fairfax lab GitHub (https://github.com/fairfaxlab).
