# Supplementary material for: CMV serostatus is associated with improved survival and delayed toxicity onset following anti-PD-1 checkpoint blockade
Source: Nat Med. 2025 Apr 23;31(7):2350–64. doi: 10.1038/s41591-025-03647-1 (PMC12283384; doi:10.1038/s41591-025-03647-1)
Supplement: Supplementary file 1 — Supplementary Fig. 1. Flow diagrams. (a) Epidemiology flow diagram. (b) Survival flow diagram. (c) irAE flow diagram. Supplementary Fig. 2. Flow gating strategy. Supplementary Fig. 3. Cohort matching tables. (a) OxCITE melanoma cohort. (b) OxCITE nonmelanoma cohort. (c) cICB-treated MM cohort with >6-month follow-up. (d) sICB-treated MM cohort with >6-month follow-up. (e) Adjuvant-treated melanoma cohort with >6-month follow-up. [file 41591_2025_3647_MOESM1_ESM.pdf]

nature medicine

Supplementary information https://doi.org/10.1038/s41591-025-03647-1

CMV serostatus is associated with improved survival and delayed toxicity onset following anti-PD-1 checkpoint blockade

In the format provided by the authors and unedited

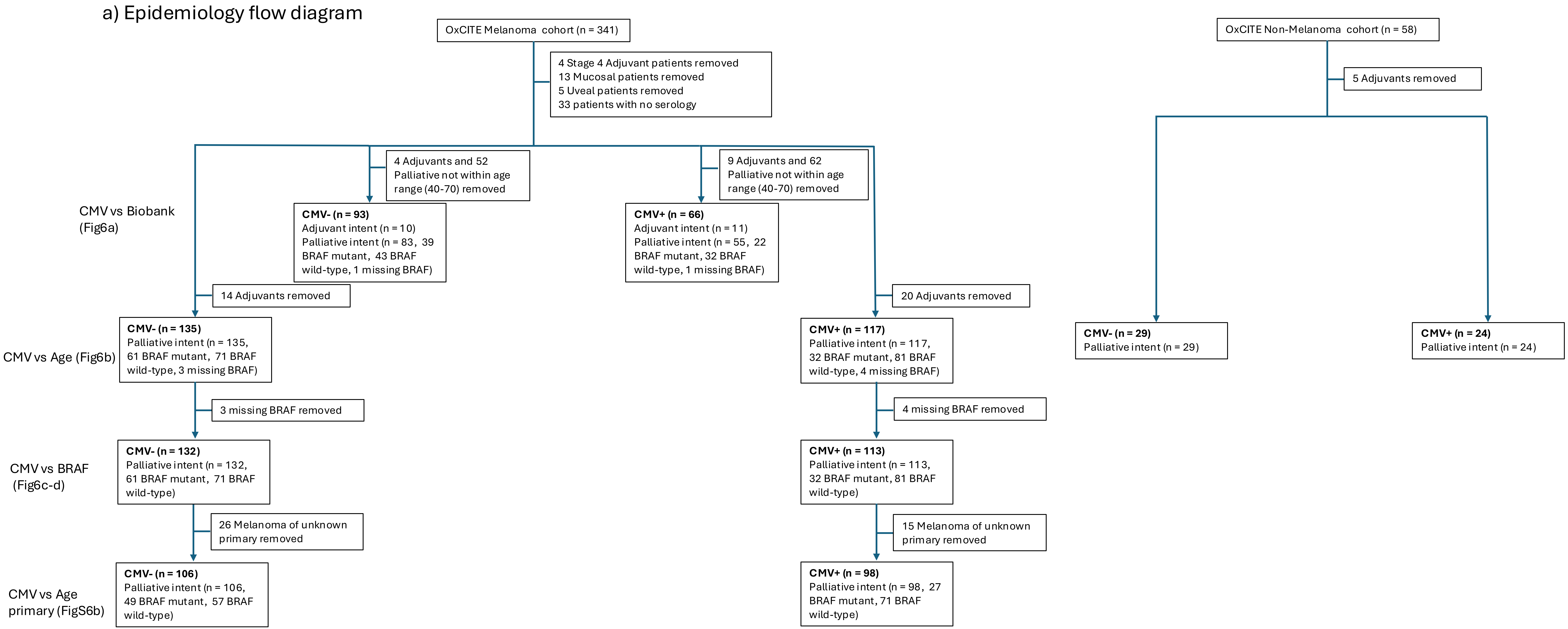

Supplementary figure 1. a) Flow diagram for patients included in epidemiology analyses

b) Survival flow diagram

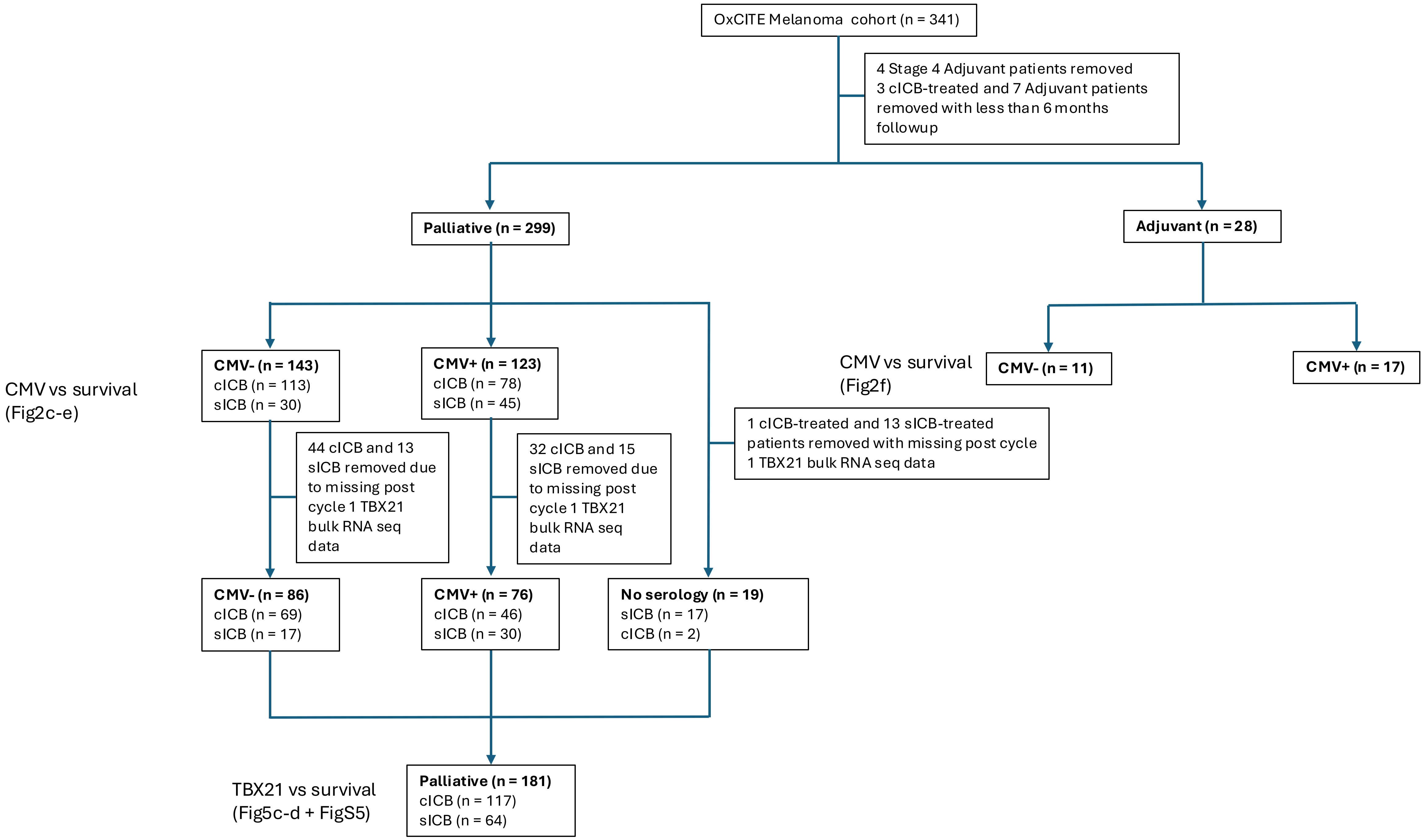

Supplementary figure 1. b) Flow diagram for patients included in survival analyses

c) irAE flow diagram

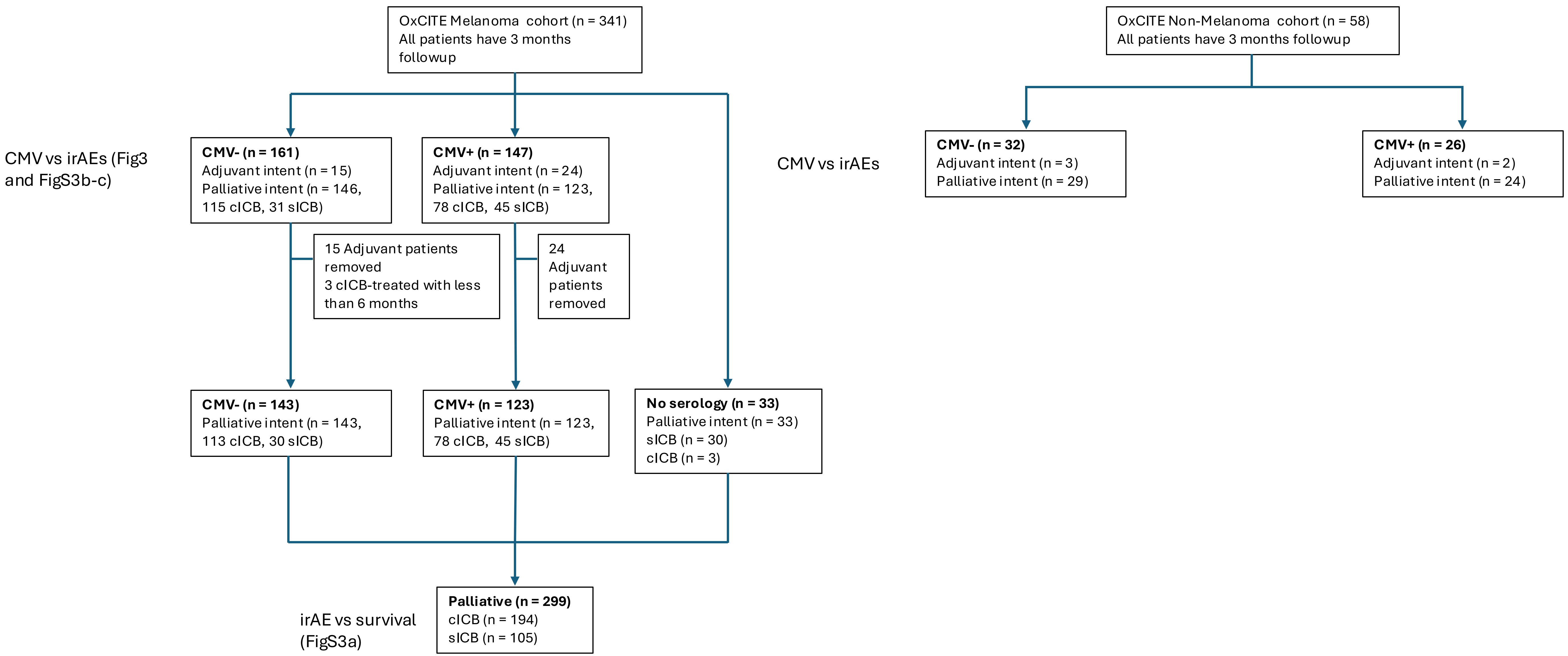

Supplementary figure 1. c) Flow diagram for patients included in irAE analyses

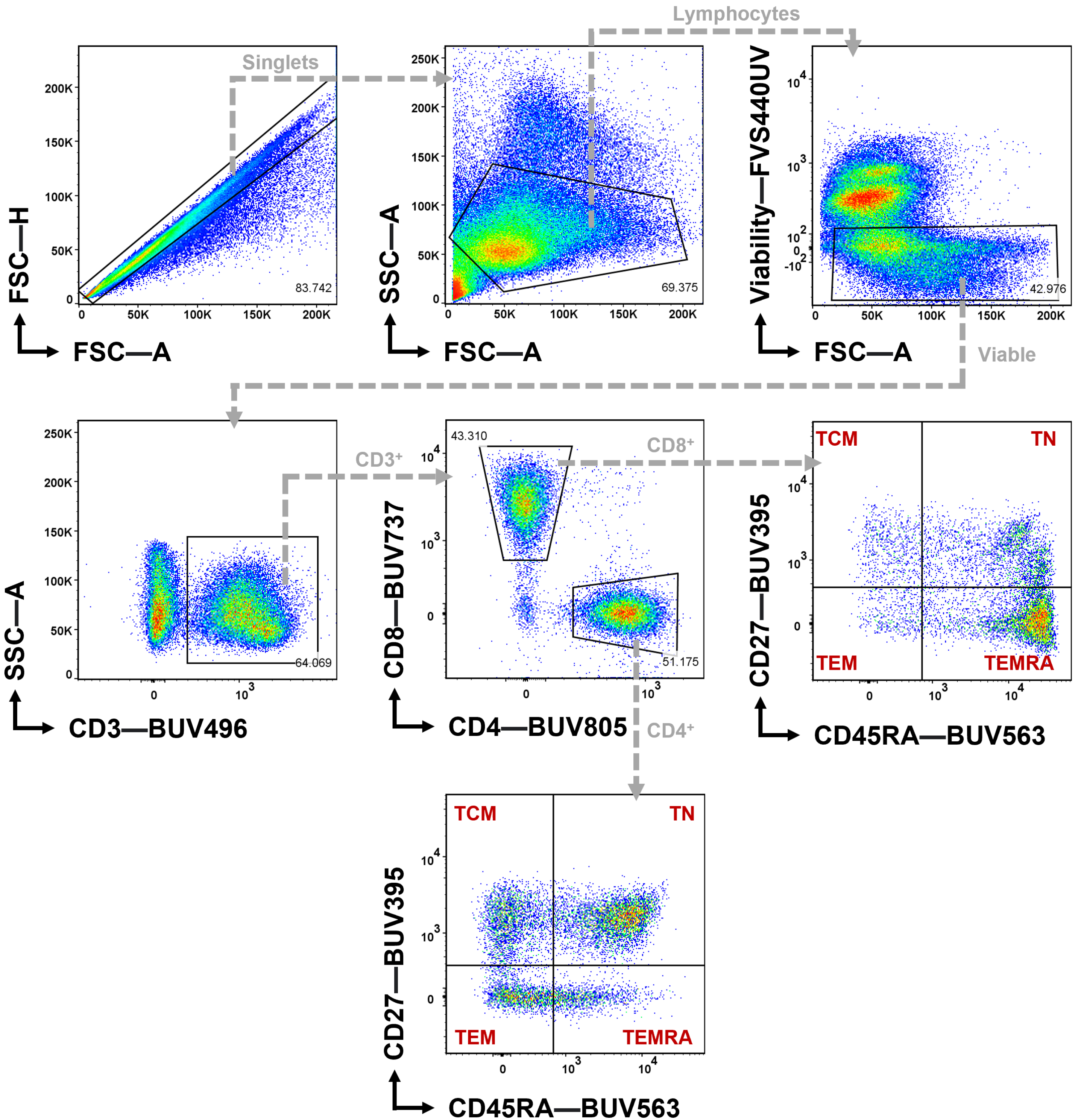

Supplementary figure 2. Flow cytometry gating strategy

a)

Characteristic	Melanoma cohort CMV- N = 161[1]	CMV+ N = 147[1]	p-value[2]
BRAF status			0.006
Mutant	66 (42%)	38 (27%)	
Wild-type	92 (58%)	105 (73%)	
Unknown	3	4	
Age	64 (55, 71)	70 (55, 77)	0.005
Sex			0.10
Female	65 (40%)	73 (50%)	
Male	96 (60%)	74 (50%)	
Treatment			<0.001
cICB	115 (71%)	78 (53%)	
sICB	46 (29%)	69 (47%)	
Treatment intent			0.065
Adjuvant	15 (9.3%)	24 (16%)	
Palliative	146 (91%)	123 (84%)	
Substage Palliative			0.4
III (unresectable)	11 (7.5%)	10 (8.1%)	
IV	0 (0%)	0 (0%)	
M1a	28 (19%)	28 (23%)	
M1b	26 (18%)	28 (23%)	
M1c	41 (28%)	35 (28%)	
M1d	40 (27%)	22 (18%)	
Unknown	15	24	
Substage Adjuvant			0.7
IIB	2 (13%)	2 (8.3%)	
IIIA	1 (6.7%)	2 (8.3%)	
IIIB	4 (27%)	5 (21%)	
IIIC	7 (47%)	10 (42%)	
IIID	1 (6.7%)	1 (4.2%)	
IV	0 (0%)	4 (17%)	
Unknown	146	123	
LDH			0.4
Normal	60 (58%)	62 (65%)	
Raised	43 (42%)	34 (35%)	
Unknown	58	51	

[1] n (%); Median (Q1, Q3)
[2] Pearson's Chi-squared test; Wilcoxon rank sum test; Fisher's exact test

b)

Characteristic	Non-Melanoma cohort CMV- N = 32[1]	CMV+ N = 26[1]	p-value[2]
Cancer			0.6
Colon	4 (13%)	6 (23%)	
cSCC	3 (9.4%)	4 (15%)	
Mesothelioma	8 (25%)	5 (19%)	
RCC	17 (53%)	11 (42%)	
Age	71 (55, 78)	69 (61, 77)	>0.9
Sex			0.4
Female	7 (22%)	8 (31%)	
Male	25 (78%)	18 (69%)	

[1] n (%); Median (Q1, Q3)
[2] Fisher's exact test; Wilcoxon rank sum test; Pearson's Chi-squared test

c)

Characteristic	cICB survival data CMV- N = 113[1]	CMV+ N = 78[1]	p-value[2]
age	61 (51, 68)	63 (51, 72)	0.11
sex			0.3
Female	46 (41%)	38 (49%)	
Male	67 (59%)	40 (51%)	
BRAF_status			0.4
Mutant	51 (46%)	29 (39%)	
Wild-type	61 (54%)	46 (61%)	
Unknown	1	3	

[1] Median (Q1, Q3); n (%)
[2] Wilcoxon rank sum test; Pearson's Chi-squared test

d)

Characteristic	sICB survival data CMV- N = 30[1]	CMV+ N = 45[1]	p-value[2]
age	79 (71, 81)	80 (75, 84)	0.055
sex			0.7
Female	14 (47%)	23 (51%)	
Male	16 (53%)	22 (49%)	
BRAF_status			0.002
Mutant	10 (36%)	3 (6.8%)	
Wild-type	18 (64%)	41 (93%)	
Unknown	2	1	

[1] Median (Q1, Q3); n (%)
[2] Wilcoxon rank sum test; Pearson's Chi-squared test

e)

Characteristic	Adjuvant survival data CMV- N = 11[1]	CMV+ N = 17[1]	p-value[2]
age	64 (56, 71)	65 (52, 72)	0.8
sex			>0.9
Female	5 (45%)	7 (41%)	
Male	6 (55%)	10 (59%)	
BRAF_status			0.7
Mutant	2 (18%)	5 (29%)	
Wild-type	9 (82%)	12 (71%)	
stage			>0.9
II	1 (9.1%)	1 (5.9%)	
III	10 (91%)	16 (94%)	

[1] Median (Q1, Q3); n (%)
[2] Wilcoxon rank sum test; Fisher's exact test

Supplementary figure 3. Cohort matching tables
