# Supplementary material for: CMV serostatus is associated with improved survival and delayed toxicity onset following anti-PD-1 checkpoint blockade
Source: Nat Med. 2025 Apr 23;31(7):2350–64. doi: 10.1038/s41591-025-03647-1 (PMC12283384; doi:10.1038/s41591-025-03647-1)
Supplement: Supplementary file 2 — Reporting Summary [file 41591_2025_3647_MOESM2_ESM.pdf]

nature portfolio

Corresponding author(s): Benjamin Fairfax

Last updated by author(s): Feb 20, 2025

Reporting Summary

Nature Portfolio wishes to improve the reproducibility of the work that we publish. This form provides structure for consistency and transparency in reporting. For further information on Nature Portfolio policies, see our Editorial Policies and the Editorial Policy Checklist.

Statistics

For all statistical analyses, confirm that the following items are present in the figure legend, table legend, main text, or Methods section.

n/a	Confirmed	
☐	☒	The exact sample size (*n*) for each experimental group/condition, given as a discrete number and unit of measurement
☐	☒	A statement on whether measurements were taken from distinct samples or whether the same sample was measured repeatedly
☐	☒	The statistical test(s) used AND whether they are one- or two-sided<br>*Only common tests should be described solely by name; describe more complex techniques in the Methods section.*
☐	☒	A description of all covariates tested
☐	☒	A description of any assumptions or corrections, such as tests of normality and adjustment for multiple comparisons
☐	☒	A full description of the statistical parameters including central tendency (e.g. means) or other basic estimates (e.g. regression coefficient) AND variation (e.g. standard deviation) or associated estimates of uncertainty (e.g. confidence intervals)
☐	☒	For null hypothesis testing, the test statistic (e.g. *F*, *t*, *r*) with confidence intervals, effect sizes, degrees of freedom and *P* value noted<br>*Give P values as exact values whenever suitable.*
☒	☐	For Bayesian analysis, information on the choice of priors and Markov chain Monte Carlo settings
☐	☒	For hierarchical and complex designs, identification of the appropriate level for tests and full reporting of outcomes
☒	☐	Estimates of effect sizes (e.g. Cohen's *d*, Pearson's *r*), indicating how they were calculated

Our web collection on statistics for biologists contains articles on many of the points above.

Software and code

Policy information about availability of computer code

Data collection	Data collection was performed by manual annotation and no specific software was used.
Data analysis	All data analysis was performed using R (version 4.4). All packages used in the analysis are described in the supplied custom code which has been made available via Code Ocean and is uploaded on the lab GitHub page: https://github.com/fairfaxlab/Cytomegalovirus-manuscript-figures. Packages used: HISAT2 (v2.2.1), HTSeq (v2.0.3), bamtools (v2.5.1), picard (v.1.105), DESeq2 (v1.40.1), XGR (v1.1.9),, pROC (v1.18.2), decoupleR (v.2.6.0), MiXCR (v3.0.13), Cellranger (v.6.0.1), scater (v1.34.0), scran (v 1.34.0), Seurat (v4), clustree (v0.5.1), SingleR (v2.8.0), MAGIC (v.2.0.3.999), ggridges (v0.5.4), survival (v3.7-0), survminer (v0.4.9), tidycmprsk (v1.0.0), ggplot (v3.5.1), MatchIt (v4.5.5), coin (v1.4-2), R (v4.4.0).

For manuscripts utilizing custom algorithms or software that are central to the research but not yet described in published literature, software must be made available to editors and reviewers. We strongly encourage code deposition in a community repository (e.g. GitHub). See the Nature Portfolio guidelines for submitting code & software for further information.

Data

Policy information about availability of data

All manuscripts must include a data availability statement. This statement should provide the following information, where applicable:

- Accession codes, unique identifiers, or web links for publicly available datasets
- A description of any restrictions on data availability
- For clinical datasets or third party data, please ensure that the statement adheres to our policy

Raw sequencing data at the individual level corresponding to anonymized patients will be made available for download from EGA (EGAD00001007942) via a Data Access Agreement between applicants and the University of Oxford 6 months from the date of publication - these data will be available for a minimum of 2 years. Requests should be addressed to benjamin.fairfax@oncology.ox.ac.uk and will be processed within the University and should take <6 weeks to action. Normalised gene expression matrices with associated minimal anonymised patient information (required to perform analyses to recreate the figures in the paper) will be available upon publication via Oxford Research Archive (https://ora.ox.ac.uk).

Research involving human participants, their data, or biological material

Policy information about studies with human participants or human data. See also policy information about sex, gender (identity/presentation), and sexual orientation and race, ethnicity and racism.

Reporting on sex and gender	Samples are described as per genetically determined biological sex at birth. Sex was used as a covariate in the analyses performed where stated.
Reporting on race, ethnicity, or other socially relevant groupings	We did not take into account ethnicity, race or socially relevant groupings in the analysis. The overwhelming majority of patients assessed were white Caucasian of Northern European ancestry.
Population characteristics	n total 399 patients were incorporated into the analyses, of which 302 had a diagnosis of metastatic melanoma, 39 had resected melanoma for which they were receiving adjuvant intent immunotherapy and 58 had mixed non-melanoma cancers treated with ICB. Across the patients with melanoma median age was 67 (IQR 55-75), for non-melanoma cancer this was 70 (IQR 57-78). For the patients with melanoma 45% were female, 26% being female for non-melanoma. All characteristics are detailed in full in Table 1,2 and Supplementary Table 6.<br>Participants who were beginning checkpoint immunotherapy for cancer were approached directly to assess whether they would consent to the Oxford Radcliffe Biobank and donate samples to the OxCITE study. Patients were selected based on NICE criteria for suitability for anti-PD1 +/- anti-CTLA-4. BRAF mutant patient OS may be influenced by either BRAFi pretreatment or post-ICB progression treatment, hence why its included as a covariate. Some patients are receiving their second line of treatment after having progressed in the adjuvant setting on anti-PD-1 (for combination-treated patients) or anti-CTLA-4 (for single agent-treated patients). Finally, patients who develop a grade 3+ irAE prior to the second cycle of treatment might not have been able to provide a post-cycle 1 blood sample, biasing the dataset towards CMV seropositive patients who have reduced incidence of early severe toxicities.
Recruitment	Participants who were beginning checkpoint immunotherapy for cancer were approached directly to assess whether they would consent to the Oxford Radcliffe Biobank and donate samples to the OxCITE study.
Ethics oversight	Informed consent was given by all patients to donate samples to the Oxford Radcliffe Biobank (Oxford Centre for Histopathology Research ethical approval reference 19/SC/0173, project nos. 16/A019, 18/A064 and 19/A114) and grant access to their routine clinical data. There was no compensation for this consent.

Note that full information on the approval of the study protocol must also be provided in the manuscript.

Field-specific reporting

Please select the one below that is the best fit for your research. If you are not sure, read the appropriate sections before making your selection.

☒ Life sciences ☐ Behavioural & social sciences ☐ Ecological, evolutionary & environmental sciences

For a reference copy of the document with all sections, see nature.com/documents/nr-reporting-summary-flat.pdf

Life sciences study design

All studies must disclose on these points even when the disclosure is negative.

Sample size	Sample size was determined by number of available patients who consented to the study who had received checkpoint immunotherapy. Three mice were used for the bulk ATACseq experiment. Each experimental group (naive, central memory and inflationary T cells) had three samples each.
Data exclusions	Overall survival analysis was only performed in patients with metastatic melanoma with >6 month follow-up, with progression free-survival also performed in patients receiving adjuvant treatment. Toxicity analysis was confined to patients who had >3months of post-treatment follow-up. Supplementary Table 6 provides information on missing data.

Replication	We sought to replicate the effects of CMV post single agent anti-PD-1 in patients receiving adjuvant anti-PD-1. Toxicity replication was described in non-melanoma patients.
Randomization	Not applicable
Blinding	Flow cytometry studies and single-cell rna sequencing were performed blind to CMV status.

Reporting for specific materials, systems and methods

We require information from authors about some types of materials, experimental systems and methods used in many studies. Here, indicate whether each material, system or method listed is relevant to your study. If you are not sure if a list item applies to your research, read the appropriate section before selecting a response.

Materials & experimental systems

n/a	Involved in the study
☐	☒ Antibodies
☒	☐ Eukaryotic cell lines
☒	☐ Palaeontology and archaeology
☒	☐ Animals and other organisms
☐	☒ Clinical data
☒	☐ Dual use research of concern
☒	☐ Plants

Methods

n/a	Involved in the study
☒	☐ ChIP-seq
☐	☒ Flow cytometry
☒	☐ MRI-based neuroimaging

Antibodies

Antibodies used	These are listed in Supplementary Table 5
Validation	As per manufacturers description

Clinical data

Policy information about clinical studies

All manuscripts should comply with the ICMJE guidelines for publication of clinical research and a completed CONSORT checklist must be included with all submissions.

Clinical trial registration	N/A
Study protocol	N/A
Data collection	Anonymised patient related data were collated from electronic health care records in accordance with ethical approval.
Outcomes	Overall survival was determined according to patient death, progression free survival according to clinician impression/ radiology. Toxicities were determined according to those recorded notes.

Plants

Seed stocks	N/A
Novel plant genotypes	N/A
Authentication	N/A

Flow Cytometry

Plots

Confirm that:

☒ The axis labels state the marker and fluorochrome used (e.g. CD4-FITC).

☒ The axis scales are clearly visible. Include numbers along axes only for bottom left plot of group (a 'group' is an analysis of identical markers).

☒ All plots are contour plots with outliers or pseudocolor plots.

☒ A numerical value for number of cells or percentage (with statistics) is provided.

Methodology

Sample preparation	Cryopreserved patient PBMCs were thawed at 37C, washed in HBSS, 1x10^6 were plated and viability staining was determined with Near IR fixable viability stain, or fixability viability stain 440UV, PBMCs were washed wtih HBSS, supplemented with 5% FBS and BD Horizon brilliant stain buffer (563794), staining for markers was performed as per antibody dilutions in Supplementary Table 5.
Instrument	Fortessa x20 or FACSymphony A5 Cell analyser
Software	FlowJo 10.7.1
Cell population abundance	As stated.
Gating strategy	Supplementary Figure 2- In brief, singletons were sorted on forward height and area scatter from PBMC. Lymphocytes were selected<br>based on viability staining and forward area scatter. CD3+ cells were selected, denoting T cells, which were then further divided into .)<br>CD4+ and CD8+ single positive populations. Major CD4+ and CD8+ subsets were determined through staining for CD27 and CD45RA. CD27+ CD45RA+ cells were defined as Naive T cells, CD27+ CD45RA- were defined as central memory, CD27- CD45RA- were defined as effector memory, and finally CD27- CD45RA+ cells were defined as TEMRA.

☒ Tick this box to confirm that a figure exemplifying the gating strategy is provided in the Supplementary Information.
